# MicroRNA‐223/NE Signaling Pathway Inhibits Lipopolysaccharide‐Induced Acute Lung Injury by Regulating Neutrophil Extracellular Traps

**DOI:** 10.1155/mi/1621608

**Published:** 2026-01-12

**Authors:** Zhengpeng Zeng, Yuexiang Qin, Xue He, Zhaoxia Tan

**Affiliations:** ^1^ Health Management Medicine Center, The Third Xiangya Hospital, Central South University, Changsha, China, csu.edu.cn

## Abstract

**Background:**

Acute lung injury (ALI) is characterized by significant neutrophil infiltration in the lungs, representing a life‐threatening condition with diverse etiologies. However, the mechanisms regulating neutrophil–alveolar epithelial interactions and the pathophysiological roles of neutrophil infiltration in ALI remain incompletely understood.

**Methods:**

A dose of 20 mg/kg lipopolysaccharide (LPS) was intratracheally instilled to induce ALI models in 10‐week‐old male microRNA‐223 knockout mice (miR‐223^−/−^) and wild‐type (WT) mice, with control group mice receiving an equal volume of phosphate‐buffered saline (PBS). After 24 h of instillation, lung tissues and peripheral blood were collected from the mice. In vivo, quantitative PCR (qPCR) measured miR‐223 and neutrophil elastase (NE) mRNA levels, while Western blot (WB), enzyme‐linked immunosorbent assay (ELISA), and hematoxylin–eosin (H&E) staining assessed neutrophil extracellular traps (NETs) markers (H3Cit, myeloperoxidase [MPO]), inflammatory cytokines (TNF‐α, IL‐1β, and IL‐6), and lung injury severity. In vitro, HL‐60‐derived neutrophil‐like cells were cocultured with alveolar epithelial cells under LPS stimulation. The roles of the miR‐223/NE/NETs axis were further investigated using the NETs inhibitor GSK484 and the NE inhibitor Sivelestat.

**Results:**

WB experiments showed an increase in NETs‐related proteins MPO and H3Cit in the lungs of WT ALI mice, with significantly enhanced expression in miR‐223^−/−^ mice. The lung injury scores and mortality rates in miR‐223^−/−^ mice were significantly exacerbated, accompanied by increased neutrophil infiltration in the lungs. Levels of inflammatory factors (TNF‐α, IL‐1β, and IL‐6) in the serum of miR‐223^−/−^ mice were significantly elevated. In vitro coculture experiments demonstrated that miR‐223 deficiency in neutrophil‐like cells augmented NETs formation and inflammatory responses, leading to increased damage to alveolar epithelial cells. However, in vivo inhibition of NETs with GSK484 or NE with Sivelestat in miR‐223^−/−^ mice significantly attenuated neutrophil infiltration, inflammation, and lung injury, and improved survival. Similarly, Sivelestat pretreatment reduced NET formation and conferred protection against ALI. Consistent with the in vivo findings, inhibition of NETs with GSK484 or NE with Sivelestat in the coculture system similarly attenuated epithelial damage and inflammatory response.

**Conclusion:**

This study reveals that the miR‐223/NE axis critically regulates NETs formation, modulating neutrophil inflammatory infiltration and neutrophil–epithelial interactions to exacerbate ALI. These findings provide potential therapeutic targets for ALI.

## 1. Introduction

Acute respiratory distress syndrome/acute lung injury (ARDS/ALI) is a clinical challenge characterized by high incidence and mortality rates, underpinned by acute diffuse lung injury, and is marked by rapid onset, swift progression, and poor prognosis [1–3]. Pneumonia, aspiration of gastric contents, mechanical ventilation, sepsis, burns, and COVID‐19 Disease 2019 (COVID‐19) are common risk factors for ARDS [3]. Despite advancements in early detection, intensive care, anti‐infective therapy, organ support, fluid management, and glucocorticoid treatment, the inhospital mortality rate for ARDS remains consistently reported at over 30% [4]. Notably, the 90‐day inhospital mortality rate for moderate to severe ARDS is as high as 43%, and survivors often face a high risk of persistent complications [5]. Over the past few decades, extensive research into pharmacological interventions for ARDS has yet to yield an effective therapeutic agent.

The prevailing clinical consensus is that the pathological basis of ARDS lies in the excessive activation, proliferation, and infiltration of neutrophils, which trigger acute pulmonary inflammation and subsequent lung injury [6]. In this process, the interaction between neutrophils and alveolar epithelial cells is a critical event in the initiation and progression of lung injury, which can be effectively modeled in vitro using differentiated HL‐60 neutrophil‐like cells cocultured with alveolar epithelial cells. Neutrophil extracellular traps (NETs), released by activated neutrophils, are net‐like complexes composed of double‐stranded DNA (dsDNA) and various substances, including myeloperoxidase (MPO), neutrophil elastase (NE), and histones [7]. The formation of NETs has a dual nature: on one hand, they can capture pathogens; on the other hand, the local high expression of NETs, induced by a large influx of neutrophils, can trigger severe immune‐inflammatory responses [8]. Due to the imbalance in the production and degradation of NETs under pathological conditions, they accumulate excessively in lung tissues and alveolar spaces, causing significant damage to lung tissue [8, 9].

MicroRNAs (miRNAs) are small noncoding RNAs that play critical roles in regulating various cellular activities, including NETosis, and are implicated in the progression of numerous diseases [10]. miRNAs exert their effects by negatively regulating gene expression, either through mRNA degradation or translational repression, thereby influencing mRNA and protein levels [11, 12]. Among these, miR‐223 is one of the most abundantly expressed miRNAs in human granulocytes and has been shown to play a pivotal role in maintaining innate immune homeostasis and regulating myeloid differentiation and granulocyte function [13]. Mice deficient in miR‐223 exhibit increased pulmonary inflammation and neutrophil infiltration, leading to heightened clinical distress following lipopolysaccharide (LPS) injection. Furthermore, miR‐223 regulates macrophage apoptosis in tuberculosis infection patients and the activation of macrophages in obesity‐related adipose tissue inflammation [14–16].

Currently, the specific mechanism by which miR‐223 regulates NETs in the pathogenesis of ARDS/ALI remains unclear, representing an important scientific question worthy of further exploration. This study aims to investigate the role of the miR‐223/NE axis as a key regulatory factor of NETs in the inflammatory injury process of ALI and to elucidate its specific mechanism, providing a basis for exploring new biological markers and therapeutic targets for ARDS/ALI.

## 2. Materials and Methods

### 2.1. Animals and Establishment of Animal Models

Wild‐type (WT) mice were purchased from Slack Corporation (Changsha, China), while miR‐223^−/−^ mice were obtained from the University of Texas Southwestern Medical Center. 8‐week‐old male C57BL/6 and miR‐223^−/−^ mice (weighing 21.68 ± 2.51 g) were randomly divided into control and ALI model groups (*n* = 6 per group). The animals were housed and managed at the Experimental Animal Center of the Third Xiangya Hospital, Central South University. LPS (Sigma–Aldrich), a major component of the cell wall of Gram‐negative bacteria, was used to induce ALI in mice. The LPS‐induced ALI model is well‐established and widely applied in high‐quality studies on ALI [17, 18]. Briefly, mice were anesthetized using 1% sodium pentobarbital to ensure a pain‐free procedure. Subsequently, a fine catheter was carefully inserted into the trachea through the glottis. A precisely measured dose of LPS (20 mg/kg) solution was then instilled directly into the lungs via this catheter. Immediately after instillation, the mice were maintained in an upright position and gently rotated to facilitate the even distribution of the LPS solution within the lungs. Control group mice received an equal volume of sterile phosphate‐buffered saline (PBS) administered in the same manner. At 24 h post‐instillation, a human ARDS‐like pathological model characterized by acute inflammation and lung parenchymal injury was successfully established. All animal procedures complied with the Guide for the Care and Use of Laboratory Animals issued by the National Institutes of Health (NIH) and were approved by the Animal Research and Ethics Committee of Central South University. Humane care was provided to all animals, and euthanasia was performed via CO_2_ inhalation.

### 2.2. Cell Culture and Treatment

HL‐60 or alveolar epithelial cells (Central South University Center for advanced research) were cultured in complete RPMI, which contained HEPES, Glutamax, and 10% fetal bovine serum (Life Technologies/Gibco, USA) at 37°C in 5% CO_2_ and passaged at least every 3–5 days to maintain cells at 2 × 10^5^ to 1.8 × 10^6^ cells/mL.

For neutrophil‐like differentiation, HL‐60 cells were resuspended at a density of 1–2 × 10^5^ cells/mL in fresh medium containing 1.25% DMSO(Sigma–Aldrich) on Day 0 to initiate the differentiation program [19]. The culture medium was replaced as needed based on cell density and medium condition during the differentiation period. For the coculture experiments, alveolar epithelial cells were seeded in the lower chambers of Transwell plates 2–3 days in advance. The differentiated HL‐60 cells were then placed in the upper chambers. Following the addition of stimulants (LPS, 100 ng/mL) or specific inhibitors, the coculture system was incubated at 37°C with 5% CO_2_ for 4–6 h. After the incubation, media and cells from both upper and lower chambers were collected separately for subsequent analysis.

### 2.3. miRNA Transfection

The successfully differentiated neutrophil‐like HL‐60 cells were collected and resuspended in fresh complete medium without DMSO. The cells were then precisely seeded at a density of 5 × 10^4^ cells per well in six‐well plates and incubated overnight (~12–16 h) at 37°C with 5% CO_2_. Working solutions were prepared by diluting either the miR‐223 mimic (Qiagen, Germany) (5 nM) or the miR‐223 inhibitor (Qiagen, Germany) (50 nM) in sterile RPMI‐1640 medium without serum or antibiotics. An appropriate volume of HiPerFect transfection reagent (Qiagen, Germany) was then added to each nucleic acid dilution, followed by immediate vortex mixing and incubation at room temperature for 10–15 min to form stable transfection complexes. After the incubation period, the transfection complexes were added dropwise and evenly to the cells, which had been refreshed with new medium. The plates were gently swirled to ensure thorough mixing and then returned to the incubator for continued culture under standard conditions (37°C, 5% CO_2_) for 24–72 h. Following transfection, cells could be either directly collected for subsequent experiments such as coculture, or the efficiency of miR‐223 overexpression or knockdown could be validated by quantitative Real‐Time PCR (qRT‐PCR), depending on the requirements of the downstream assays.

### 2.4. Cell Viability Assay

After treatment under various conditions, alveolar epithelial cells were collected. The old culture medium was discarded, and the cells were gently rinsed twice with pre‐warmed PBS. Subsequently, 900 μL of serum‐free basal medium and 110 μL of CCK‐8 solution (Dojindo, ShangHai, China) were added to each well, followed by gentle shaking of the plate to ensure thorough mixing. The plate was then placed in a 37°C, 5% CO_2_ incubator and protected from light for 1–4 h, with periodic observation. Once the solution color turned orange‐yellow and sufficient chromogenic development was achieved, the absorbance of each well was measured at a wavelength of 450 nm using a microplate reader.

### 2.5. qRT‐PCR

Total RNA was extracted from lung tissues using TRIzol reagent (Takara, Dalian, China) following the manufacturer’s instructions. RNA purity and concentration were measured using a NanoDrop spectrophotometer (Agilent Technologies, USA). Subsequently, single‐stranded cDNA was synthesized using HiScript II QRT SuperMix for quantitative PCR (qPCR, R223‐01, Vazyme, China) according to the manufacturer’s protocol. Finally, relative quantification was performed using ChamQ SYBR Color qPCR Master Mix (Q431‐02, Vazyme, China) on a Fast 7500 Real‐Time PCR System (Applied Biosystems, USA). GAPDH served as the endogenous reference, and the 2^−ΔΔCT^ method was used to calculate the fold changes in target gene expression. The qRT‐PCR primers used in this study were synthesized by Sangon Biotech (Shanghai, China) with the following sequences:

Mouse MicroRNA‐223 Forward:5′‐TGTCAGTTTGTCAAATACCCCA‐3′,
Mouse MicroRNA‐223 Reverse:5′‐CAGTGCAGGGTCCGAGGTATT‐3′,
Mouse NE Forward:5′‐GCTGGAGGTGCTGCTGTCTA‐3′,
Mouse NE Reverse:5′‐TGGTAGCCACAGAGCCATTG‐3′,
Mouse GAPDH Forward: 5′‐CTGGAACCGCATCATCGTGGAG‐3′,
Mouse GAPDH Reverse:5′‐CCTGATGATCCCAAATTCATCAAAATAG‐3′.

### 2.6. Enzyme‐Linked Immunosorbent Assay (ELISA)

Commercial ELISA kits (CusaBio, China) were used to quantify serum levels of TNF‐α, IL‐6, and IL‐1β following the manufacturer’s instructions. Briefly, microplates were coated with capture antibodies overnight at 4°C and then blocked with PBS containing 4% bovine serum albumin (BSA) and 5% sucrose. Serum samples and standards were added in duplicate and incubated at 37°C for 1 h. After washing, detection antibodies were added and incubated for another hour. Following thorough washing, streptavidin‐conjugated horseradish peroxidase (HRP) was added and incubated at 37°C for 30 min. The reaction was developed using TMB substrate (incubated at 37°C for 10 min in the dark) and stopped with H_2_SO_4_. Absorbance was measured at 450 nm using a microplate reader (SPECTROstar Nano, BGM/LABTECH).

### 2.7. Protein Extraction and Western Blot (WB) Analysis

Cells and lung tissues were homogenized in RIPA lysis buffer on ice for 30 min. Protein concentrations were determined using a bicinchoninic acid (BCA) protein assay kit (Wellbio, Changsha, China). Equal amounts of protein were separated by 12% SDS‐PAGE and transferred onto polyvinylidene difluoride (PVDF) membranes. The membranes were then incubated with primary antibodies (H3Cit and MPO from Abcam; GAPDH from Proteintech) and corresponding secondary antibodies, followed by enhanced chemiluminescence (ECL) detection. Protein bands were quantified using a ChemiDoc XRS + imaging system (Bio‐Rad, Hercules, CA, USA), with GAPDH serving as the loading control.

### 2.8. Cell Counting in Bronchoalveolar Lavage Fluid (BALF)

Mice were euthanized 24 h after LPS instillation. The trachea was lavaged three times with ice–cold PBS to collect BALF cells. An equal volume of BALF and 0.2% glacial acetic acid was mixed, and a small aliquot was loaded onto a hemocytometer for total cell counting under a microscope. The BALF was centrifuged at 6000 rpm for 10 min at 4°C to separate the supernatant and cell pellet. The pellet was resuspended in PBS, smeared onto slides, and stained with Giemsa–Wright stain for microscopic examination. Cell counting was performed in a single‐blind manner by the same investigator.

### 2.9. Pharmacological Inhibitor Treatment in Mice

8‐week‐old male C57BL/6J mice were intraperitoneally injected with either Sivelestat (a NE inhibitor, 150 mg/kg, dissolved in 5% DMSO, 40% PEG300, 5% Tween 80, and 50% ddH_2_O; Selleck, Houston, TX) or GSK484 (a NETosis inhibitor, 20 mg/kg, dissolved in PBS; Sigma–Aldrich) 24 h before ALI induction.

### 2.10. Lung Histology

The same lung lobes from mice in different groups were preserved in 4% paraformaldehyde for 24 h. Then, the lung tissue was embedded in paraffin wax, sliced, and stained with hematoxylin–eosin (H&E). The thickness of the tissue sections was 3–4 μm. Lung histology sections were observed via microscope, and evaluation of ALI was performed by a single researcher independently. Alveolar edema, pulmonary hemorrhage, leukocyte infiltration, and alveolar septal thickening were evaluated, and each item had four ranks, namely normal (score of 0), mild (score of 1), moderate (score of 2), and severe (score of 3), based on which the assessment of ALI was calculated.

### 2.11. Statistical Analysis

Data are described as the mean ± standard deviation (SD) and were analyzed using GraphPad Prim 8. For comparisons between two datasets, Student’s *t* test was applied if they were distributed normally; otherwise, the Mann–Whitney test was applied. For more than two groups, one‐way ANOVA was applied, and Tukey analysis was used as a post hoc test. A value of *p*  < 0.05 was considered statistically significant. Asterisks in the figures represent the following:  ^∗^ *p*  < 0.05;  ^∗∗^ *p*  < 0.01;  ^∗∗∗^ *p*  < 0.001. Values are from ≥6 separate experiments.

## 3. Results

### 3.1. The Deficiency of MiR‐223 Increases the Formation of NETs in the Lungs of LPS‐induced ALI Mice and Exacerbates Lung Injury in Mice

We first studied whether miR‐223 contributes to the pathogenesis of ALI in mice by comparing the phenotypes of miR‐223 knockout (miR‐223^−/−^) and WT controls. Compared to the WT control group, the lung injury in miR‐223^−/−^ mice was significantly aggravated, with a marked increase in inflammatory cell infiltration and alveolar hemorrhage observed in H&E‐stained lung sections (Figure 1A,B). Considering that miR‐223 is one of the most abundant miRNAs in neutrophils, our results show that the abundance of miR‐223 in the lungs of LPS‐induced WT mice was significantly enriched by about 15 times compared to the control group, while there was no expression in miR‐223^−/−^ mice (Figure 1C). Moreover, under LPS intervention, the levels of NE RNA and protein in miR‐223^−/−^ mice were significantly higher than those in WT mice (Figure 1 D,E,H). Next, we assessed whether lung of LPS‐induced ALI mice underwent NET formation. Interestingly, WB analysis revealed that compared to the WT control group, the levels of specific markers of NET formation, H3Cit and MPO, were significantly increased in the lungs of LPS‐induced miR‐223^−/−^ mice (Figure 1E–G).

Figure 1MiR‐223 deficiency enhances NETs formation and exacerbates lung injury in LPS‐induced ALI mouse model. (A,B) H&E‐stained lung sections demonstrating markedly aggravated lung injury in miR‐223^−/−^ mice, characterized by increased inflammatory cell infiltration and alveolar hemorrhage compared to WT controls. Scale bar: 20 μm. (C) qRT‐PCR analysis showing miR‐223 expression was significantly enriched ~15‐fold in lungs of LPS‐induced wild‐type mice compared to controls, while no expression was detected in miR‐223^−/−^ mice. (D,E) Under LPS challenge, both mRNA and protein levels of neutrophil elastase (NE) were significantly elevated in lungs of miR‐223^−/−^ mice compared to WT mice. (E–G) Western blot analysis revealed significantly increased expression of NET formation‐specific markers H3Cit and MPO in lungs of LPS‐induced miR‐223^−/−^ mice compared to WT control group. (H) Western blot analysis demonstrated a significant upregulation in the protein expression of NE in the lungs of LPS‐induced miR‐223^−/−^ mice relative to the WT control group. Data are expressed as means ± SEM. *n* = 6.  ^∗^ *p*  < 0.05,  ^∗∗^ *p*  < 0.01, ^∗∗∗^ *p*  < 0.01,  ^∗∗∗∗^ *p* < 0.01.

**Figure:**
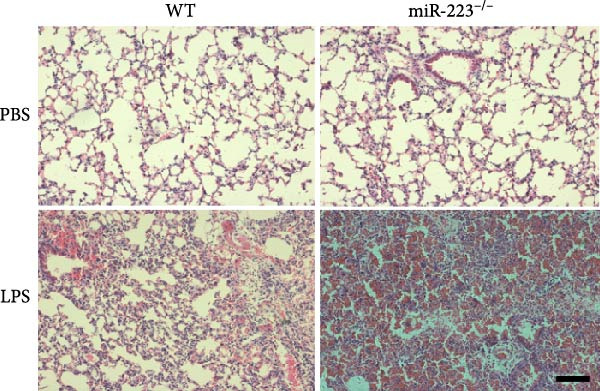
(A)

**Figure:**
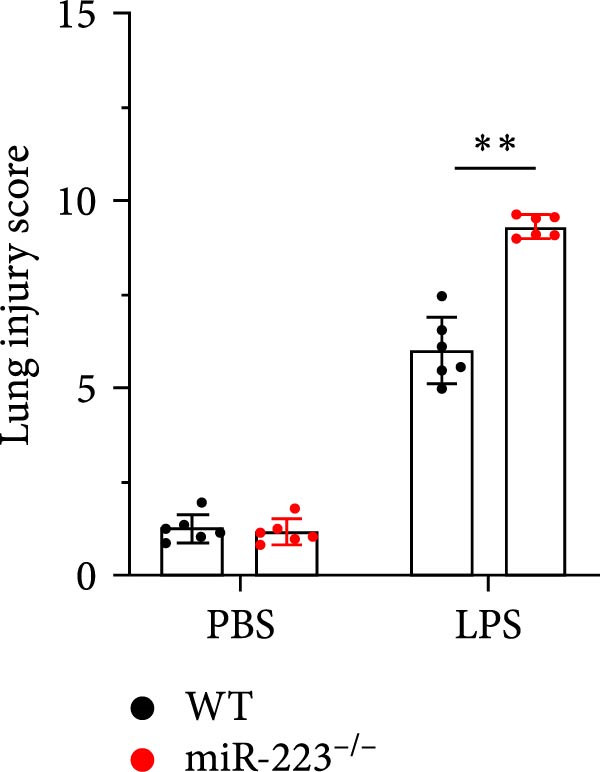
(B)

**Figure:**
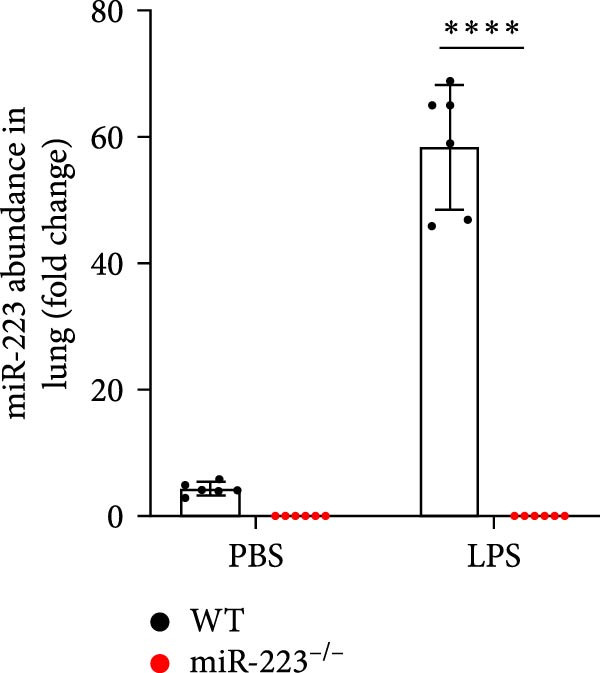
(C)

**Figure:**
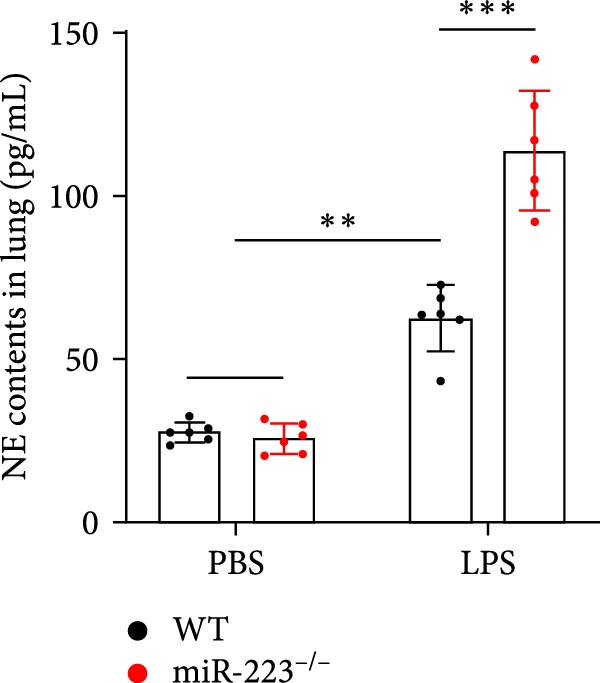
(D)

**Figure:**
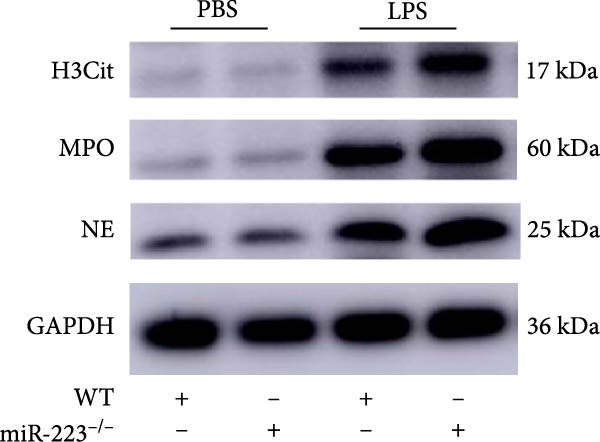
(E)

**Figure:**
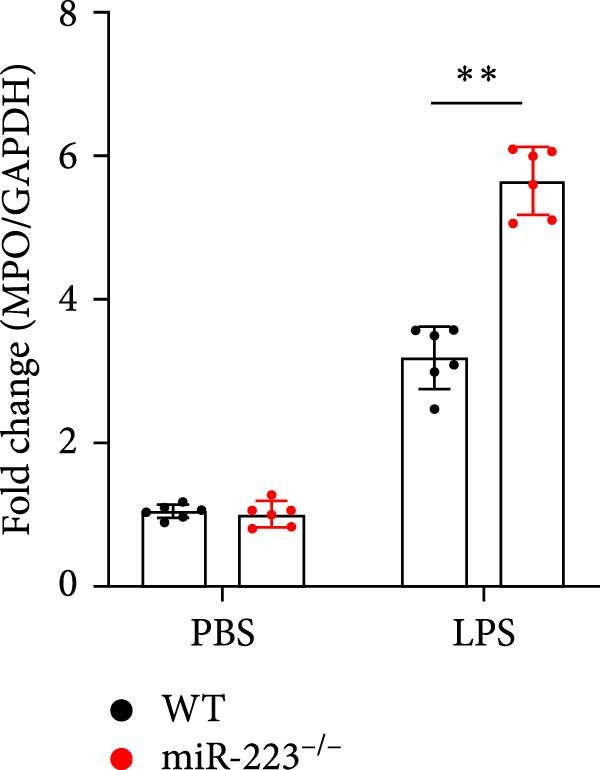
(F)

**Figure:**
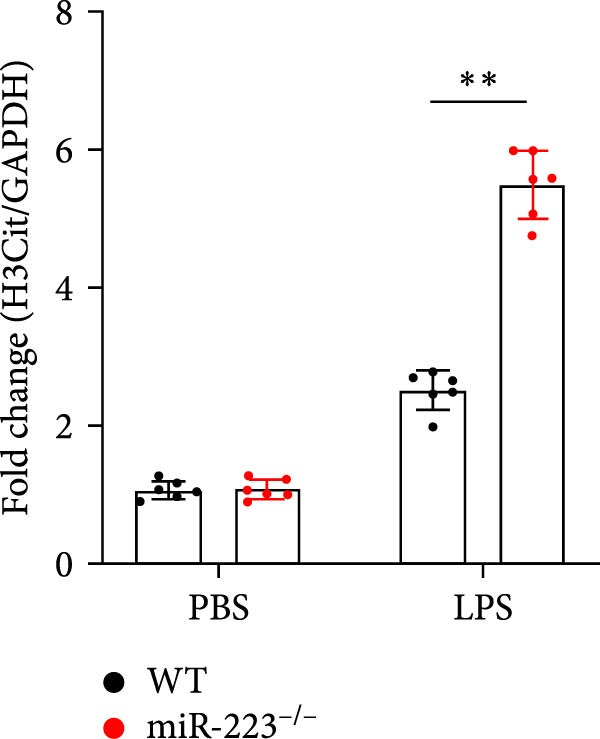
(G)

**Figure:**
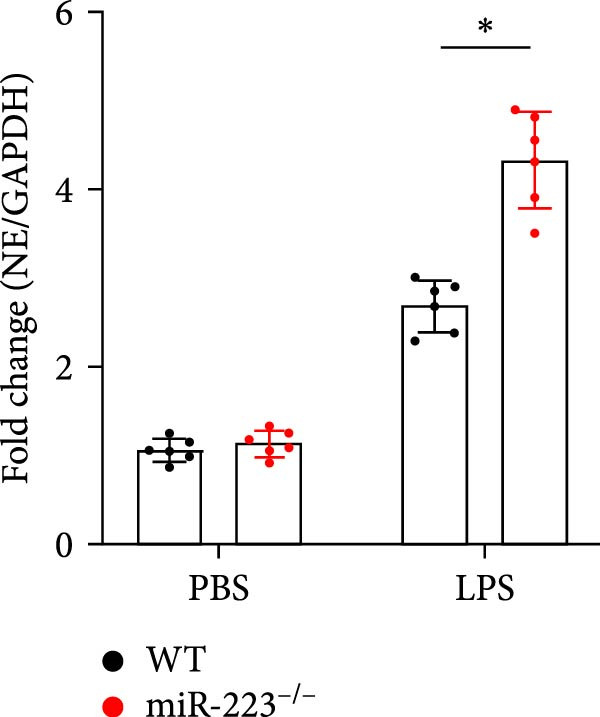
(H)

### 3.2. MiR‐223^−/−^ Leads to Increased Inflammatory Levels and Reduced Survival Rates in LPS‐Induced ALI Mice

Compared to the WT control group, the abundance of TNF‐α, IL‐6, and IL‐1β in the serum of miR‐223^−/−^ is significantly increased (Figure 2A–C). Moreover, the protein content and cell count in the BALF of miR‐223^−/−^ are markedly higher than those in the control group (Figure 2D,E). Similarly, under LPS intervention, the survival rate of miR‐223^−/−^ is significantly lower than that of WT mice (Figure 2F). In summary, the deficiency of miR‐223 significantly enhances the susceptibility of mice to LPS‐induced ALI.

Figure 2MiR‐223 deficiency exacerbates systemic inflammation and reduces survival in LPS‐induced ALI mice. (A–C) Serum levels of TNF‐α, IL‐6, and IL‐1β were significantly elevated in miR‐223^−/−^ mice compared to WT controls following LPS challenge. (D–E) Bronchoalveolar lavage fluid from miR‐223^−/−^ mice showed markedly increased total protein concentration and cellularity relative to WT controls. (F) Survival curves demonstrated significantly higher mortality in miR‐223^−/−^ mice compared to WT mice under LPS induction. Data are expressed as means ± SEM. *n* = 6.  ^∗^ *p*  < 0.05,  ^∗∗^ *p*  < 0.01, ^∗∗∗^ *p*  < 0.01.

**Figure:**
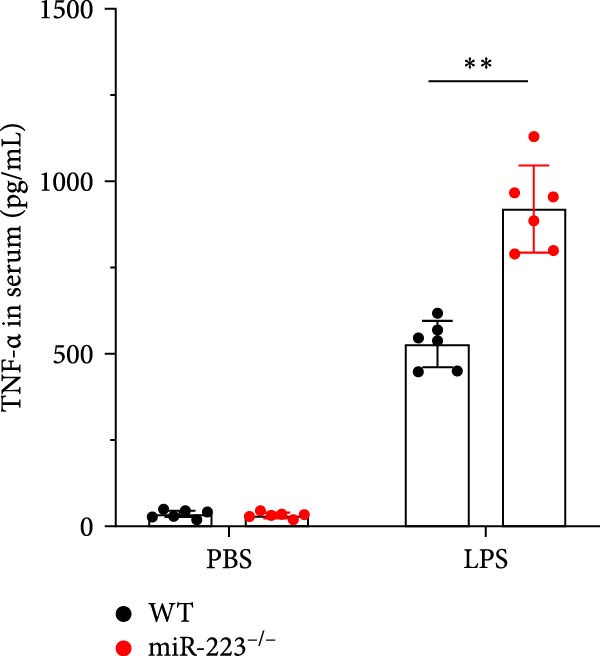
(A)

**Figure:**
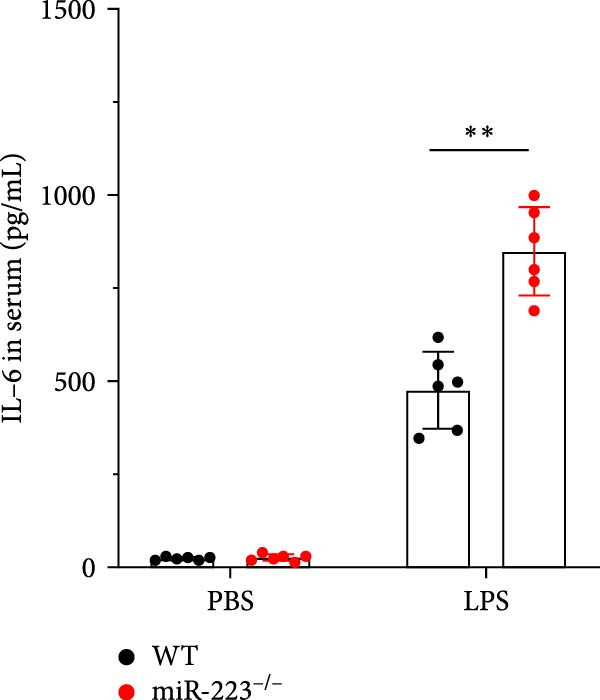
(B)

**Figure:**
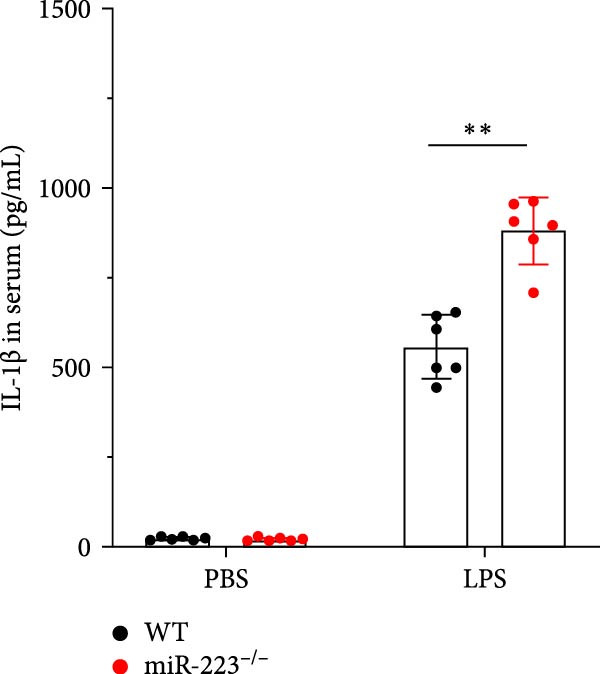
(C)

**Figure:**
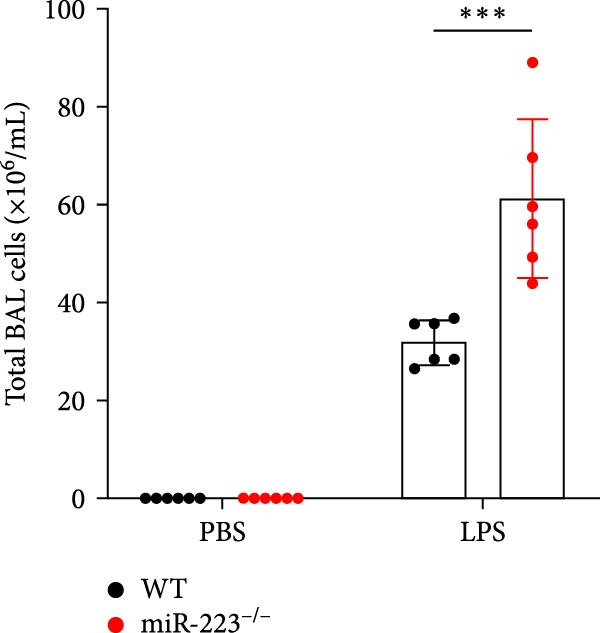
(D)

**Figure:**
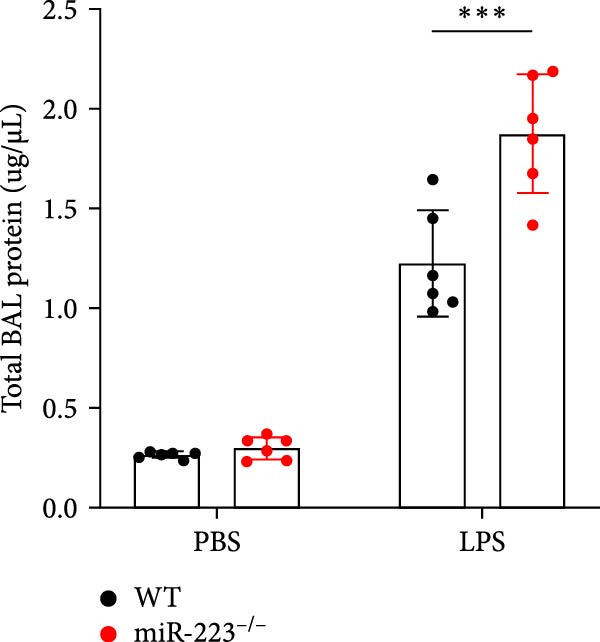
(E)

**Figure:**
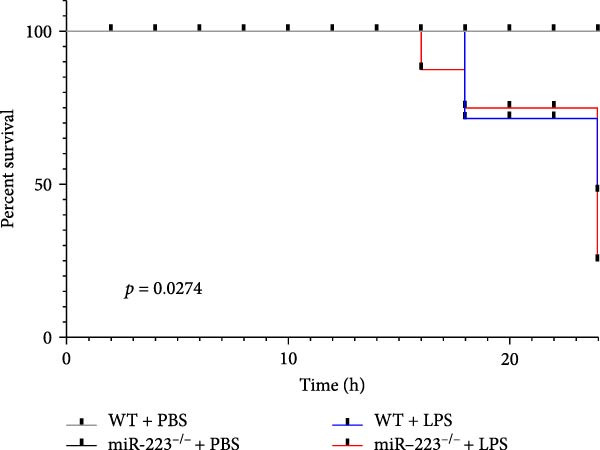
(F)

### 3.3. The Inhibition of NE by Sivelestat Alleviates ALI in WT Mice

We subsequently tested the pharmacological effect of Sivelestat, a selective, reversible, and competitive NE inhibitor, on ALI mice. Sivelestat pretreatment was administered 24 h prior to LPS treatment. H&E staining revealed that lung injury in mice pretreated with Sivelestat was significantly reduced compared to the LPS‐treated control group (Figure 3A,B). Notably, H&E staining demonstrated that Sivelestat‐mediated NE inhibition significantly reduced neutrophil infiltration in ALI, which was corroborated by assessing the abundance of the neutrophil markers MPO and H3Cit in the lungs (Figure 3C–E). Similarly, the elevation of serum TNF‐α, IL‐1β, and IL‐6 levels induced by LPS was markedly diminished in the Sivelestat pretreatment group (Figure 3F–H). Consistent with this anti‐inflammatory effect, BALF analysis revealed substantially reduced total protein concentration and cellularity in Sivelestat‐pretreated mice compared to LPS‐only controls (Figure 3 I,J). However, this treatment did not reverse the mortality rate in mice (Figure 3K). Due to the significantly increased sensitivity of NE levels in the lungs associated with ALI, we used Sivelestat pretreatment to inhibit NE in WT mice, and the results showed that this treatment provided protective effects against ALI. Therefore, blocking NE protects mice from the effects of ALI, at least partially by inhibiting neutrophil infiltration in the lungs.

Figure 3Sivelestat‐mediated neutrophil elastase (NE) inhibition attenuates acute lung injury in WT mice. (A,B) H&E‐stained lung sections demonstrating significantly reduced lung injury in Sivelestat‐pretreated mice compared to LPS‐only treated controls. (C–E) Western blot analysis showing Sivelestat‐mediated NE inhibition significantly decreased neutrophil infiltration in ALI, as evidenced by reduced expression of neutrophil markers MPO and H3Cit in lung tissues. (F–H) Serum levels of TNF‐α, IL‐1β, and IL‐6 were markedly suppressed in Sivelestat‐pretreated group compared to LPS‐induced controls. (I,J) Bronchoalveolar lavage fluid analysis revealed substantially reduced total protein concentration and cellularity in Sivelestat‐pretreated mice compared to LPS‐only controls. (K) Survival curve analysis indicated that Sivelestat pretreatment did not significantly reverse mortality rates in LPS‐challenged mice. Data are expressed as means ± SEM. *n* = 6.  ^∗^ *p*  < 0.05,  ^∗∗^ *p*  < 0.01, ^∗∗∗^ *p*  < 0.01.

**Figure:**
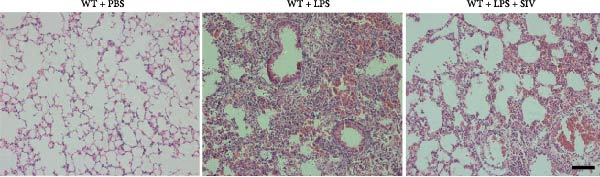
(A)

**Figure:**
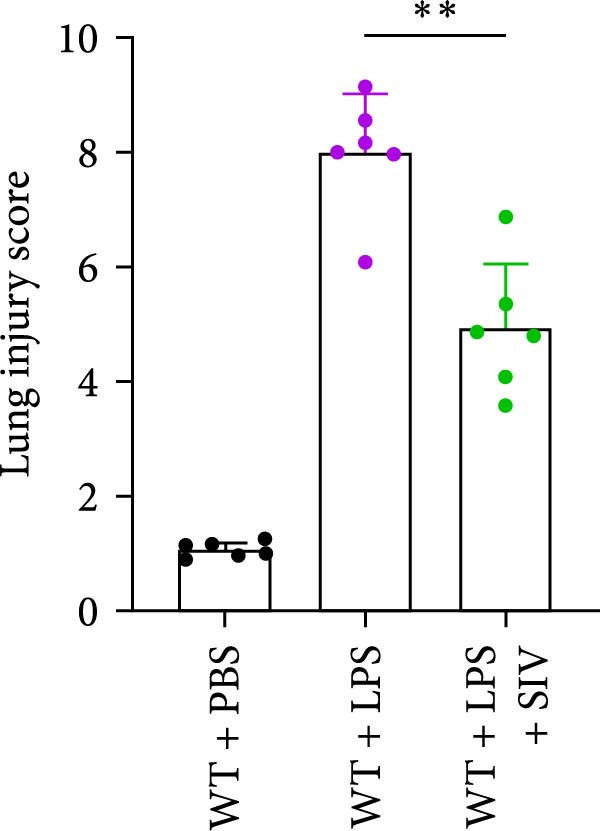
(B)

**Figure:**
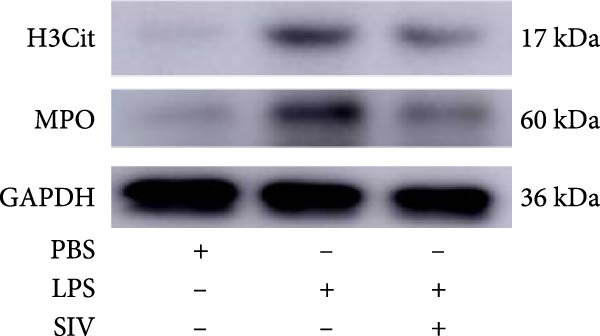
(C)

**Figure:**
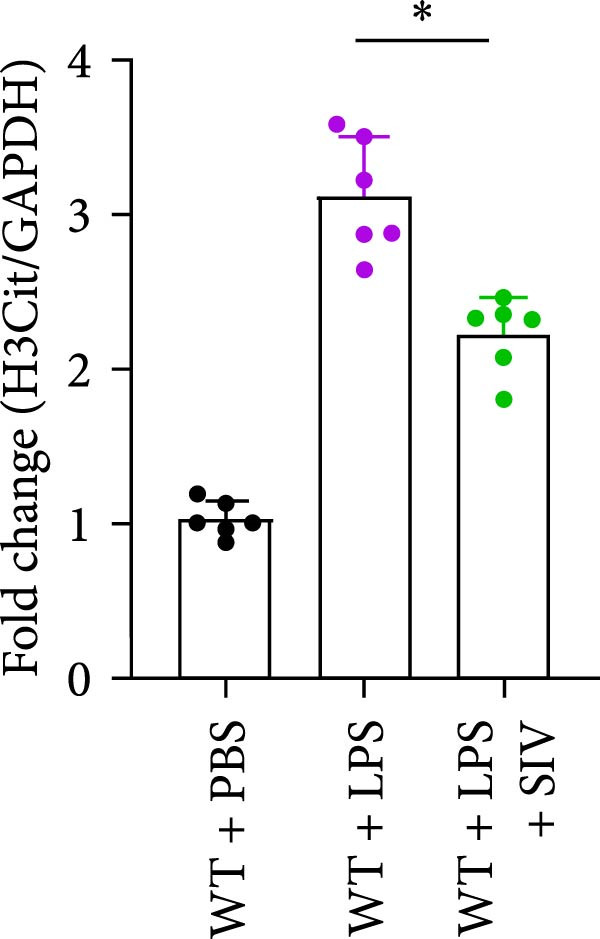
(D)

**Figure:**
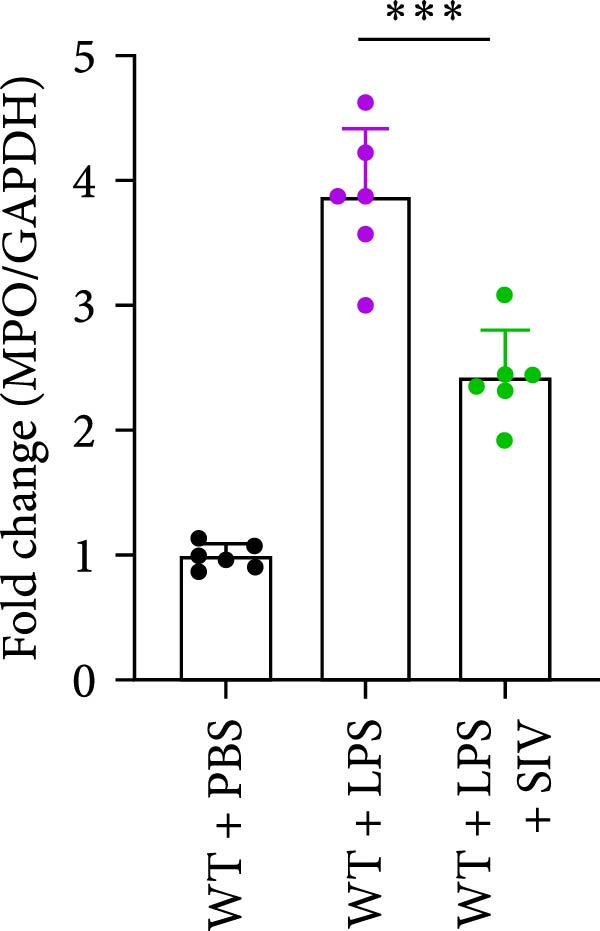
(E)

**Figure:**
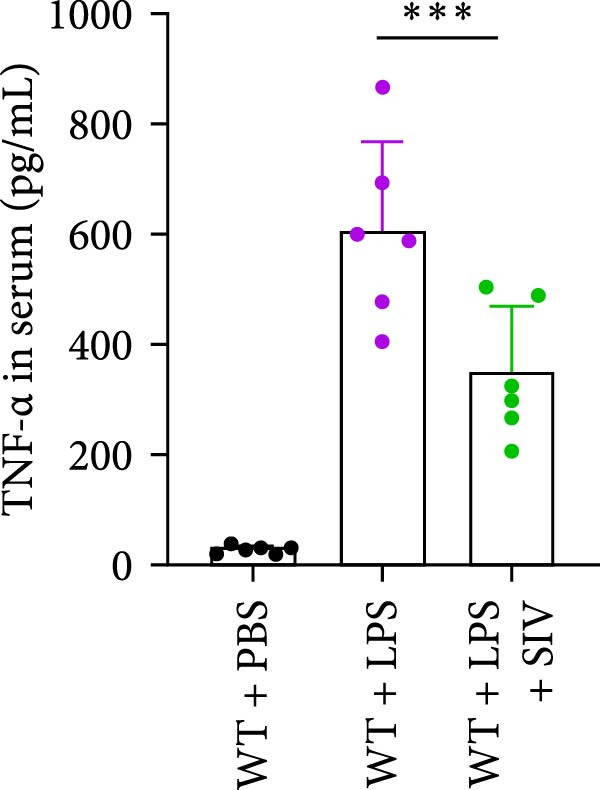
(F)

**Figure:**
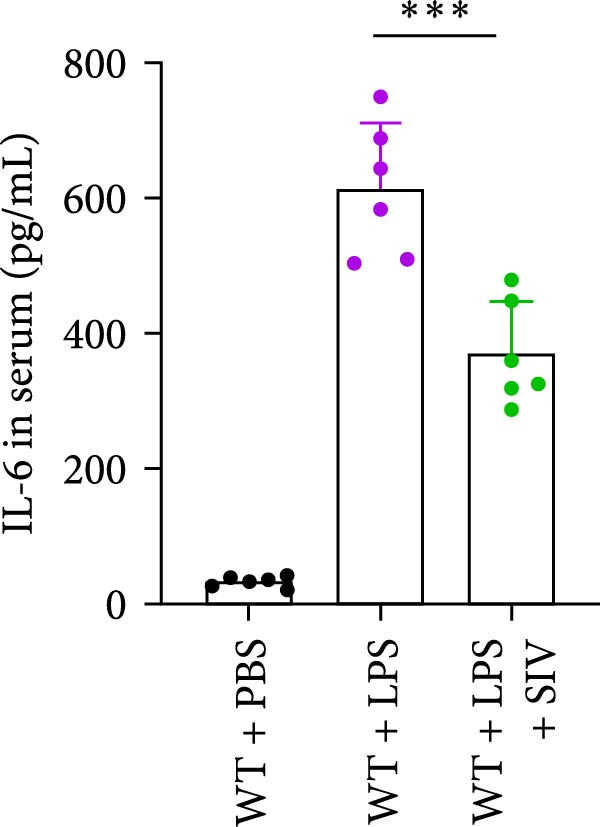
(G)

**Figure:**
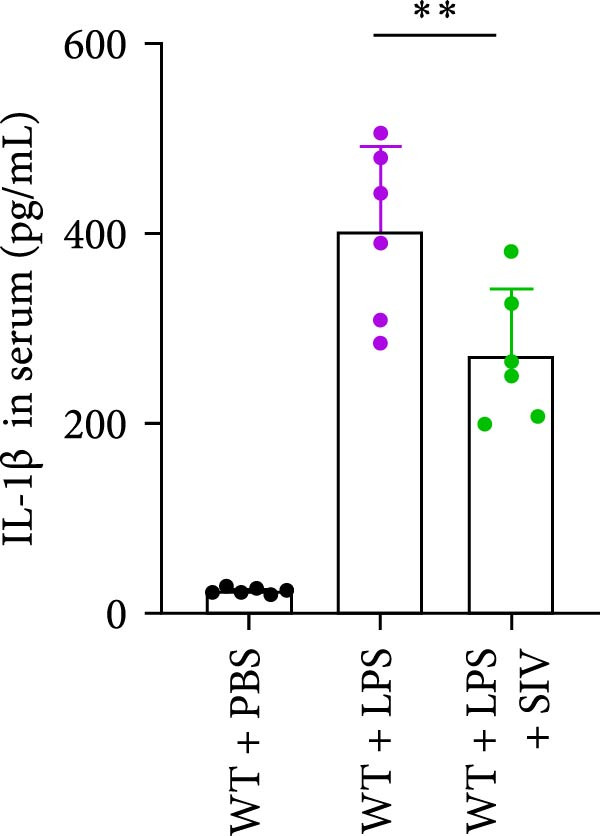
(H)

**Figure:**
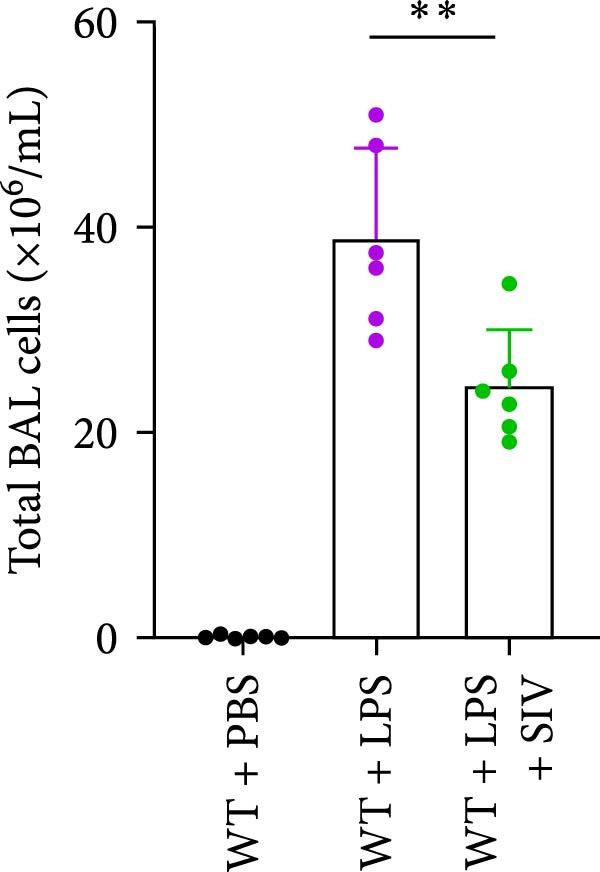
(I)

**Figure:**
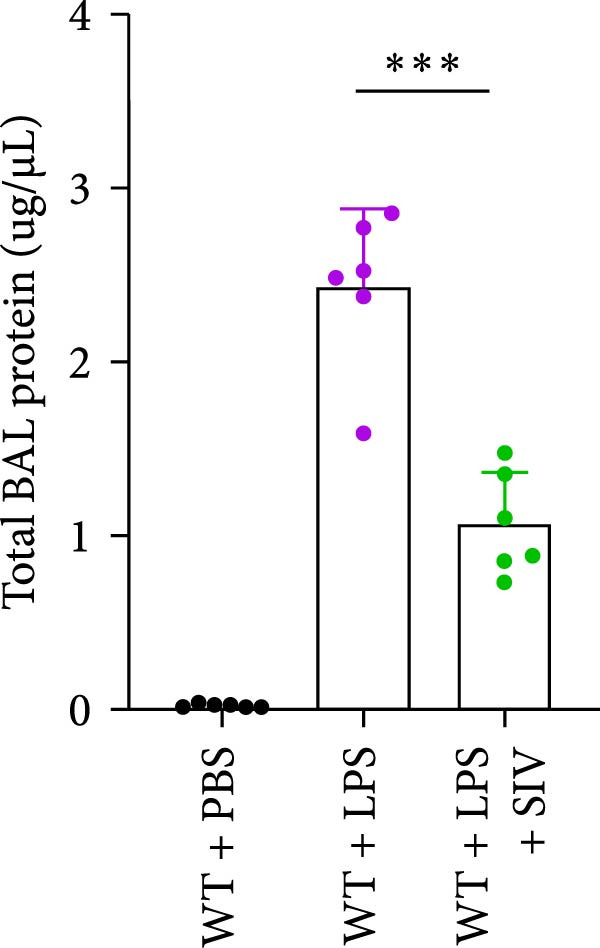
(J)

**Figure:**
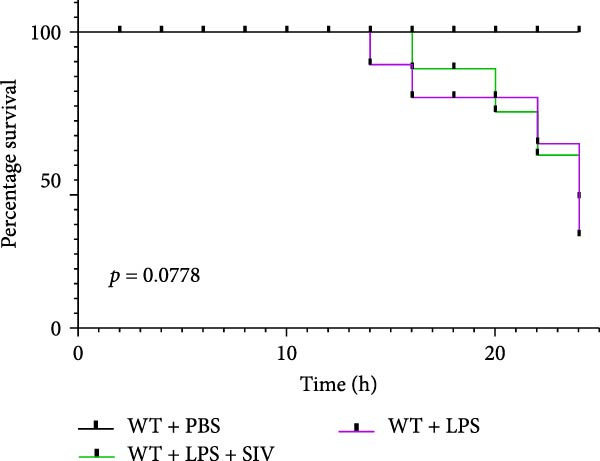
(K)

### 3.4. GSK484‐Mediated NETosis Blockade Protects WT Mice From ALI Response Attenuation

To determine whether NETs associated with ALI in the lungs play a critical role in disease progression, we used a selective and effective inhibitor, GSK484, to block protein arginine deiminase 4 (PAD4), a key requirement for NET formation. Mice were intraperitoneally injected with GSK484 at a dose of 20 mg/kg body weight 24 h prior to LPS intervention. In terms of treatment, compared to the control group, the extensive lung damage induced by LPS in GSK484 pretreated mice was significantly reduced, as observed in H&E‐stained lung sections (Figure 4A,B). In the GSK484 treatment group, neutrophil infiltration in ALI‐induced lungs was also significantly diminished, which can be confirmed by measuring the neutrophil markers MPO and H3Cit (Figure 4C–E). GSK484 pretreatment resulted in a significant decrease in serum levels of TNF‐α, IL‐1β, and IL‐6 in LPS‐induced WT mice, and the protein content and cell count in the BALF of WT mice were significantly lower than those in the control group (Figure 4F–J). Additionally, in GSK484 pretreated mice, the high mortality rate associated with the lethal dose of LPS in the control group was significantly reversed (Figure 4K). These data indicate that GSK484 can reduce the formation of ALI‐induced pulmonary NETosis and exert protective effects against ALI and its associated mortality by suppressing inflammatory levels.

Figure 4GSK484‐mediated NETosis blockade confers protection against ALI in WT mice. (A,B) H&E‐stained lung sections showing significant attenuation of LPS‐induced extensive lung damage in GSK484‐pretreated mice compared to controls. (C–E) Western blot analysis demonstrating GSK484 treatment effectively suppressed NETs formation in LPS‐challenged lung tissues, as evidenced by reduced expression of neutrophil markers MPO and H3Cit. (F–H) Serum pro‐inflammatory cytokine levels (TNF‐α, IL‐1β, and IL‐6) were significantly decreased in GSK484‐pretreated group following LPS induction. (I,J) Bronchoalveolar lavage fluid analysis revealed substantially reduced total protein concentration and cellularity in GSK484‐pretreated mice compared to LPS‐only controls. (K) Survival curves showing GSK484 pretreatment significantly reversed the high mortality rate associated with lethal‐dose LPS challenge in control group. Data are expressed as means ± SEM. *n* = 6.  ^∗^ *p*  < 0.05,  ^∗∗^ *p*  < 0.01, ^∗∗∗^ *p*  < 0.01.

**Figure:**
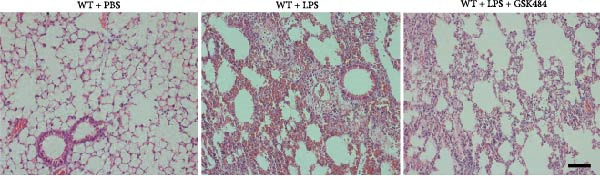
(A)

**Figure:**
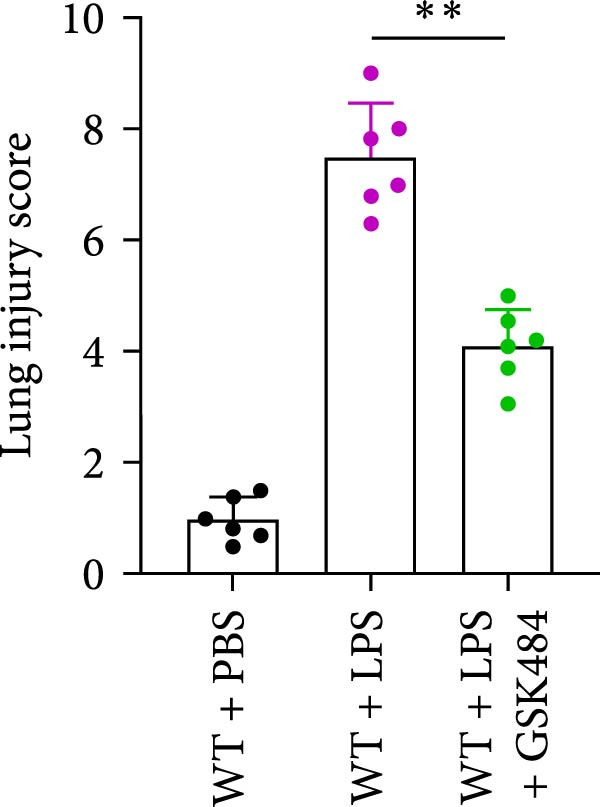
(B)

**Figure:**
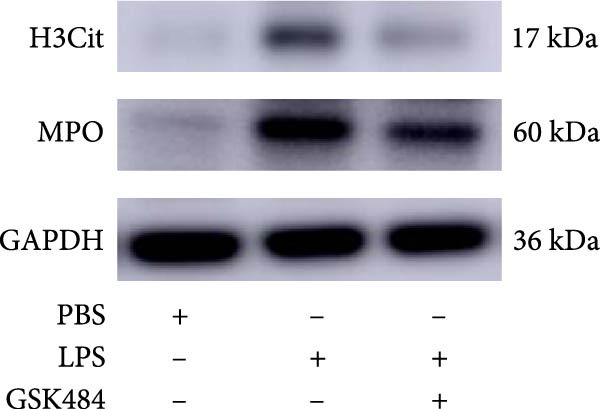
(C)

**Figure:**
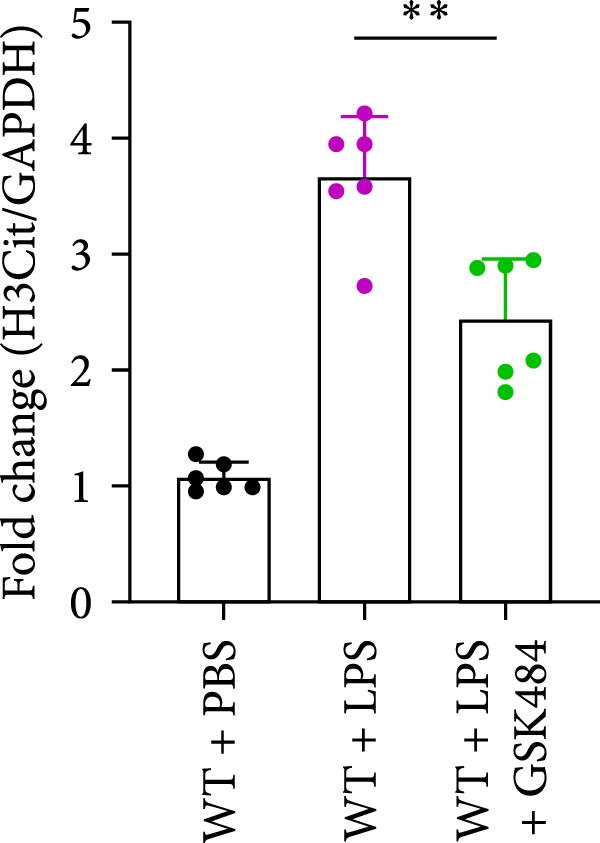
(D)

**Figure:**
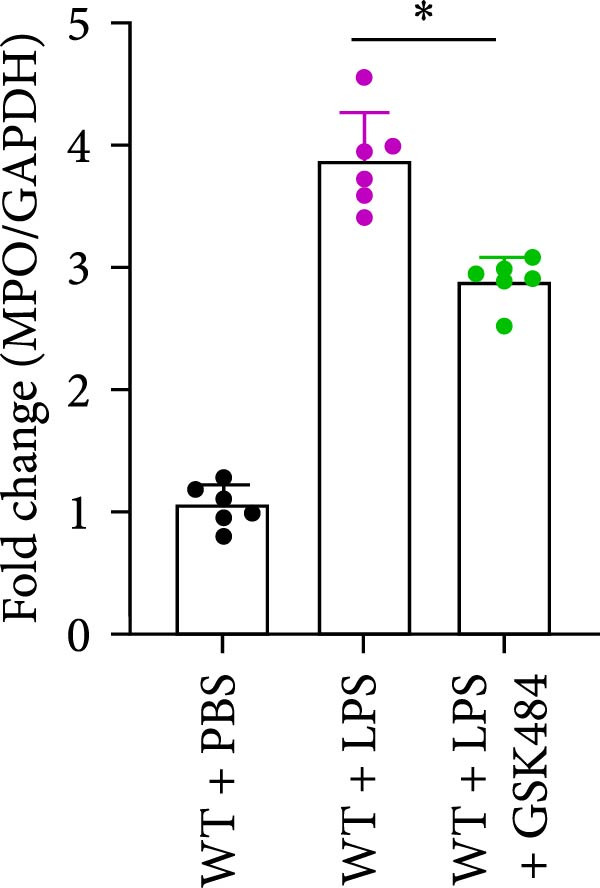
(E)

**Figure:**
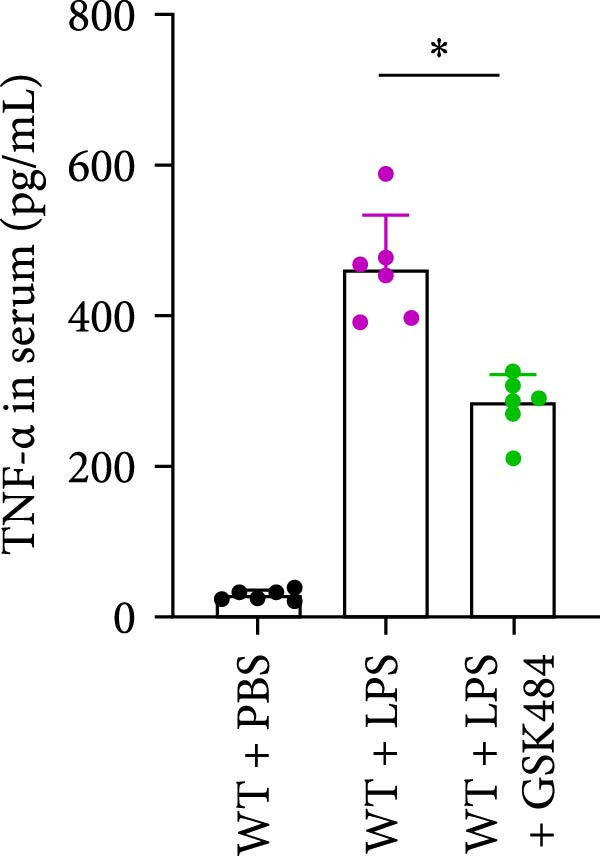
(F)

**Figure:**
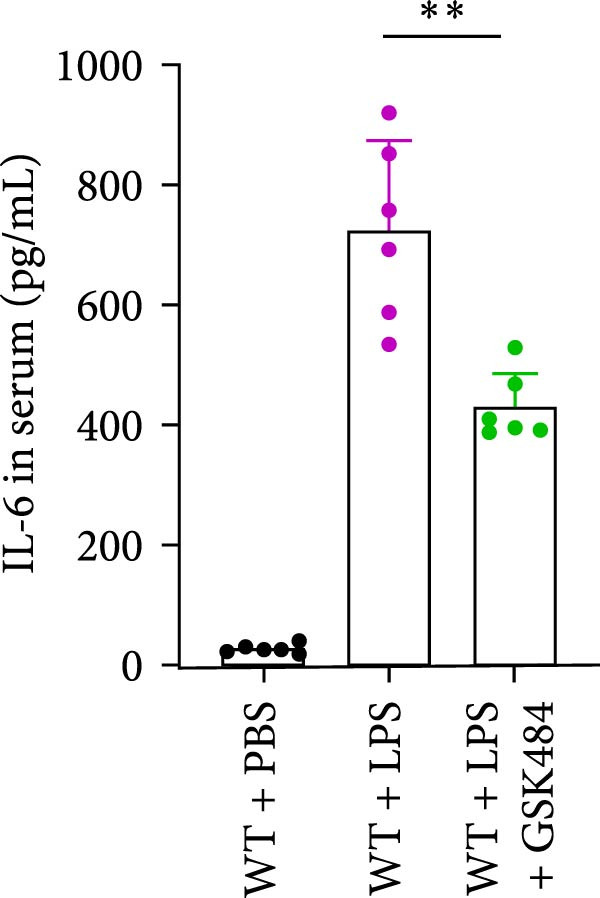
(G)

**Figure:**
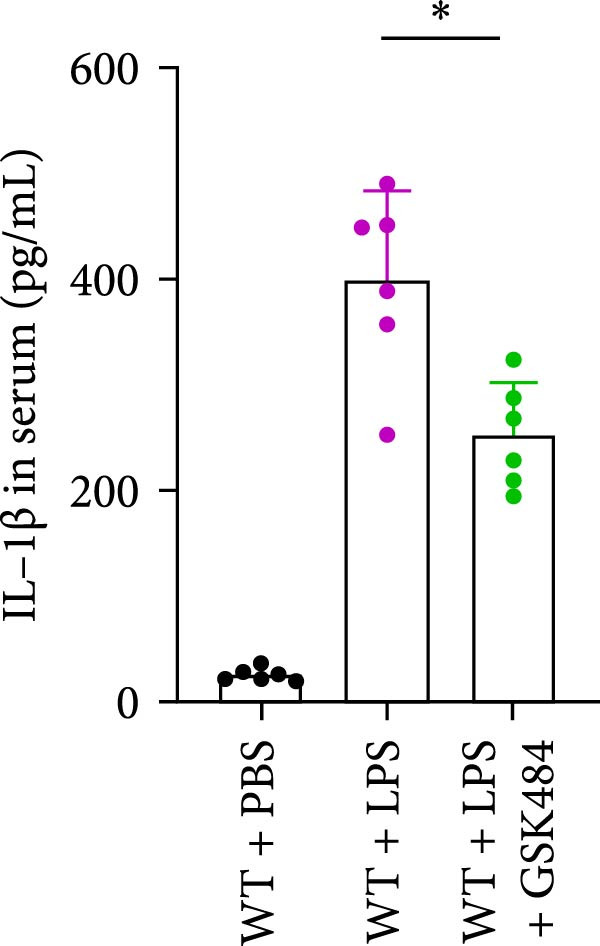
(H)

**Figure:**
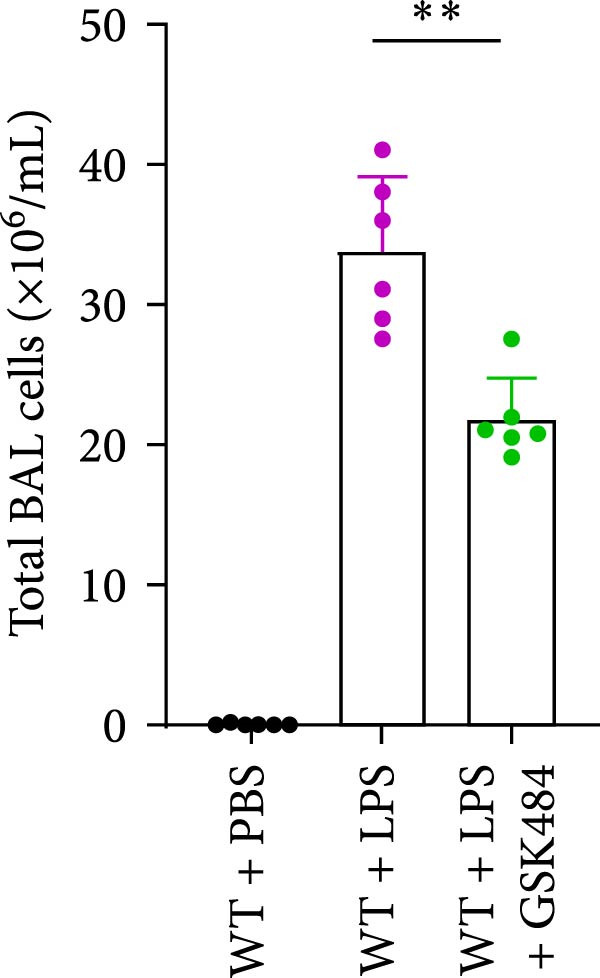
(I)

**Figure:**
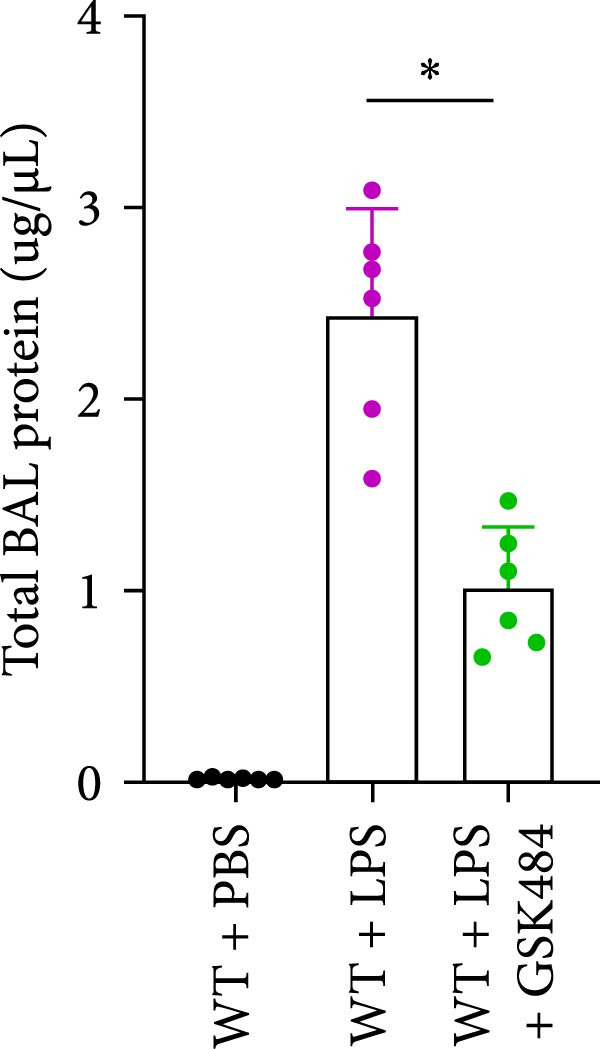
(J)

**Figure:**
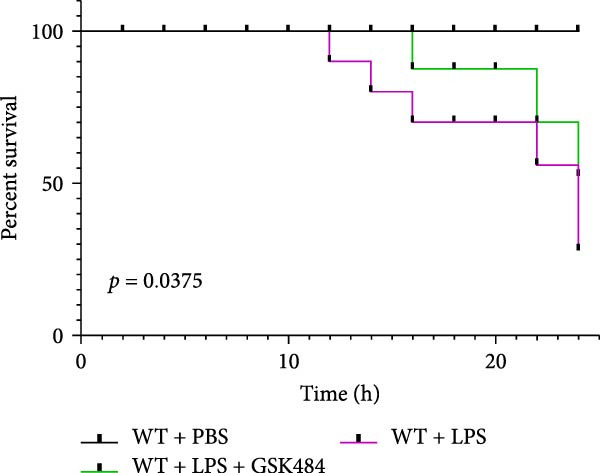
(K)

### 3.5. GSK484‐Mediated NETosis Blockade Protects miR‐223^−/−^ Mice From ALI Response Attenuation

To elucidate the relationship between miR‐223 and NETosis in the pathophysiological processes of ALI, we compared the response of miR‐223^−/−^ mice to GSK484 treatment with the control group. GSK484‐mediated NETosis inhibition exerted beneficial effects in miR‐223^−/−^ mice, which can be demonstrated by the histopathology of lung tissues and the levels of inflammatory factors (Figure 5A–K). Nevertheless, compared to the WT control group, the degree of beneficial effects of GSK484 pretreatment in miR‐223^−/−^ mice was greater, primarily due to the increased severity of lung injury in miR‐223^−/−^ mice under baseline conditions of vehicle treatment.

Figure 5GSK484‐mediated NETosis inhibition ameliorates ALI in miR‐223^−/−^ mice. (A,B) H&E‐stained lung sections demonstrating improved lung histopathology in GSK484‐pretreated miR‐223^−/−^ mice. (C–E) Western blot analysis showing significant reduction in MPO and H3Cit protein expression in lung tissues of GSK484‐treated miR‐223^−/−^ mice. (F–H) Serum levels of inflammatory mediators (TNF‐α, IL‐1β, and IL‐6) were significantly reduced in GSK484‐pretreated miR‐223^−/−^ mice. (I,J) Bronchoalveolar lavage fluid analysis revealed markedly decreased total protein concentration and cellularity in the treatment group. (K) Survival curves showing improved survival rate in GSK484‐pretreated miR‐223^−/−^ mice compared to vehicle controls.  ^∗^ *p* < 0.05,  ^∗∗^ *p* < 0.01.

**Figure:**
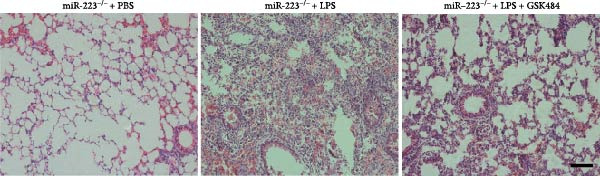
(A)

**Figure:**
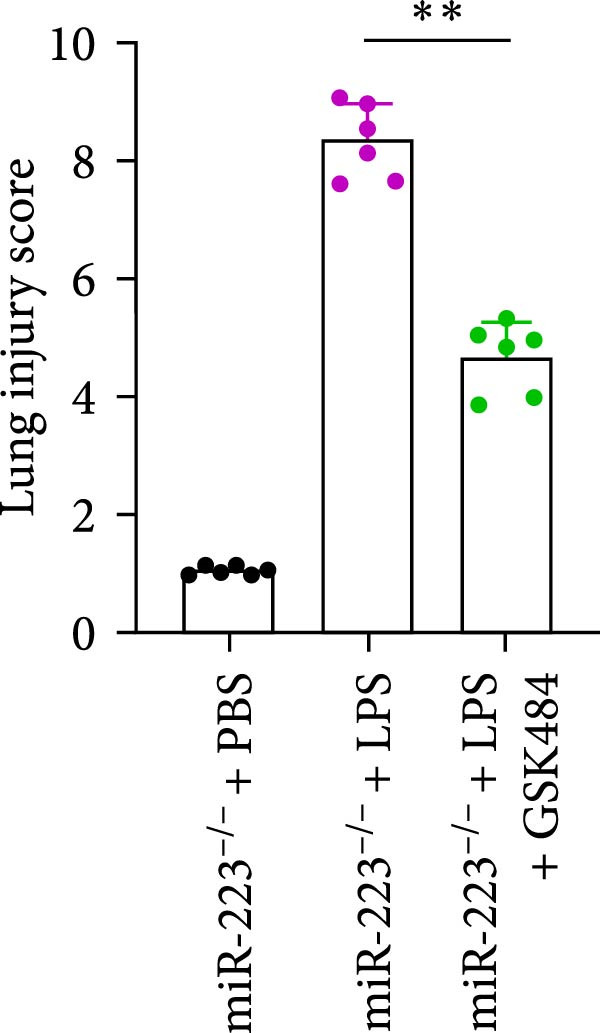
(B)

**Figure:**
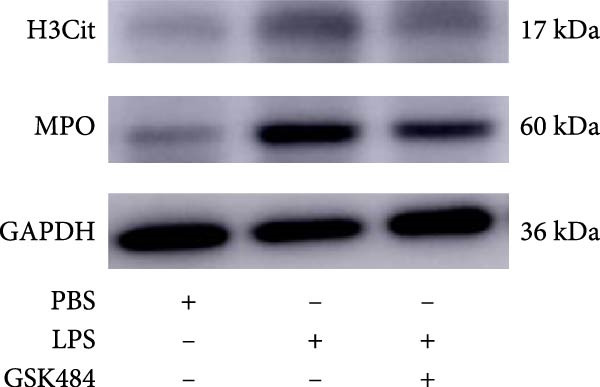
(C)

**Figure:**
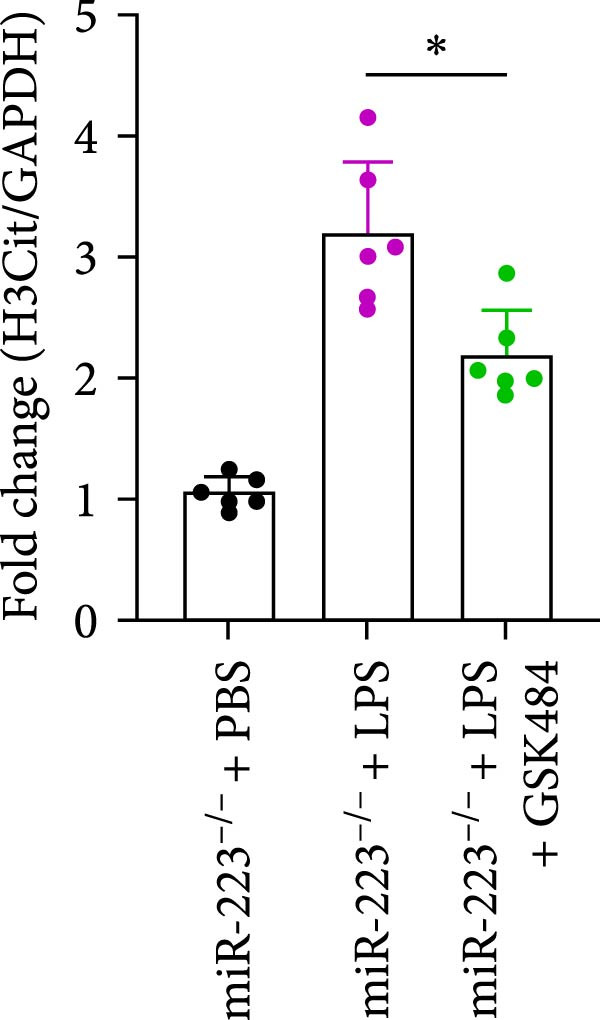
(D)

**Figure:**
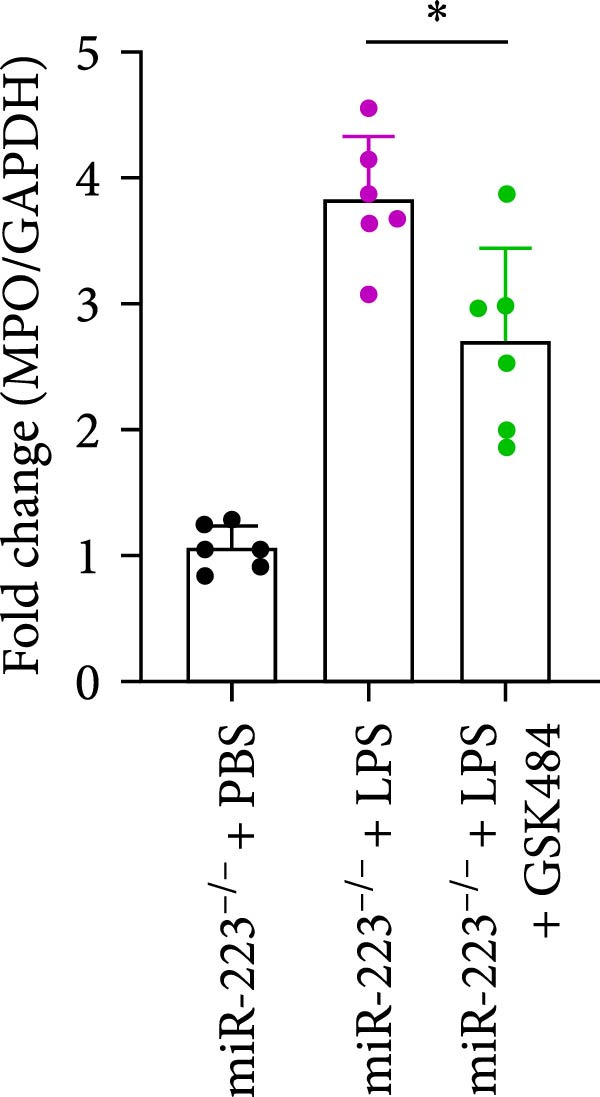
(E)

**Figure:**
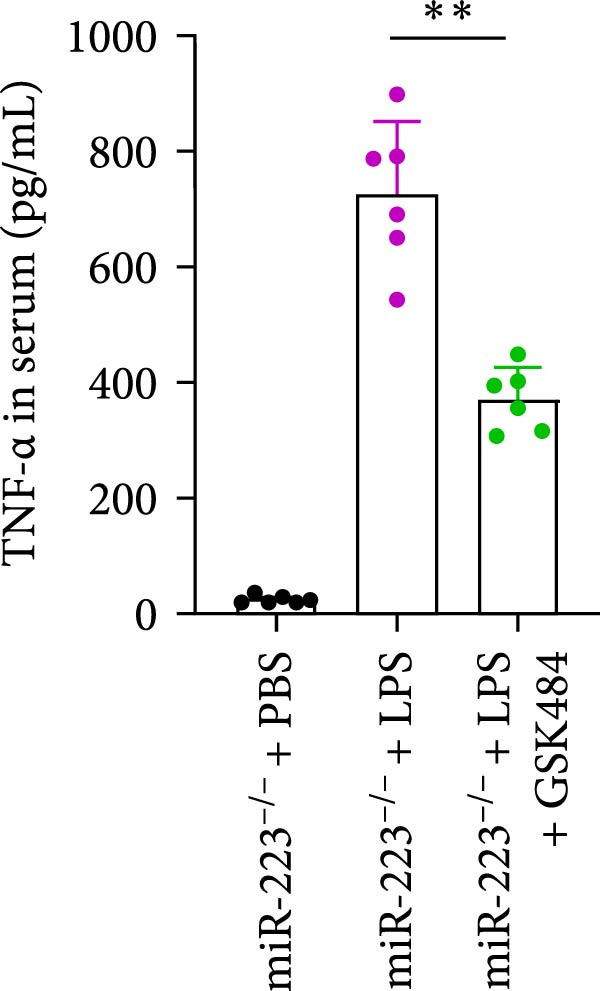
(F)

**Figure:**
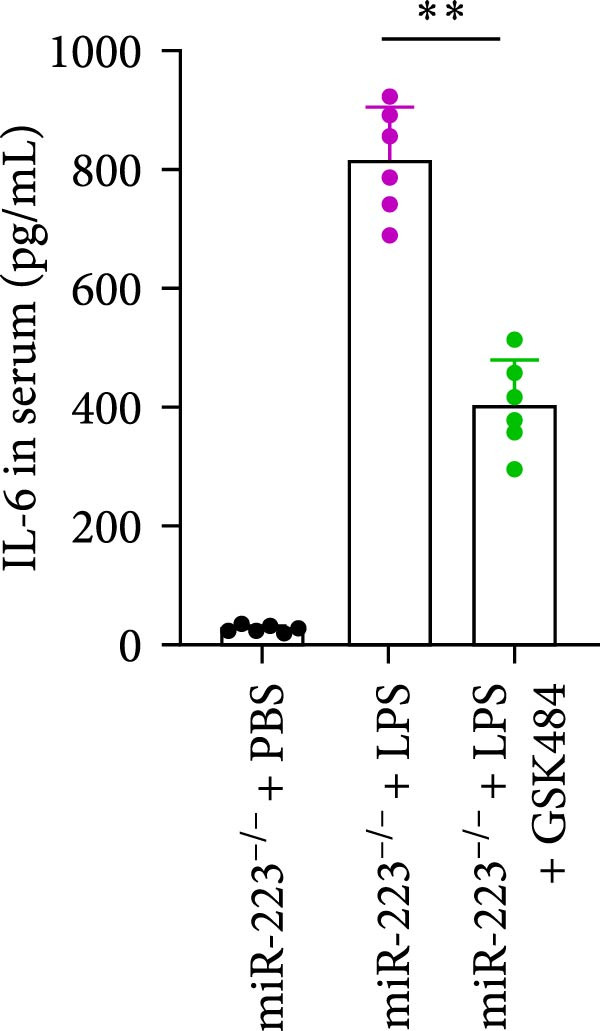
(G)

**Figure:**
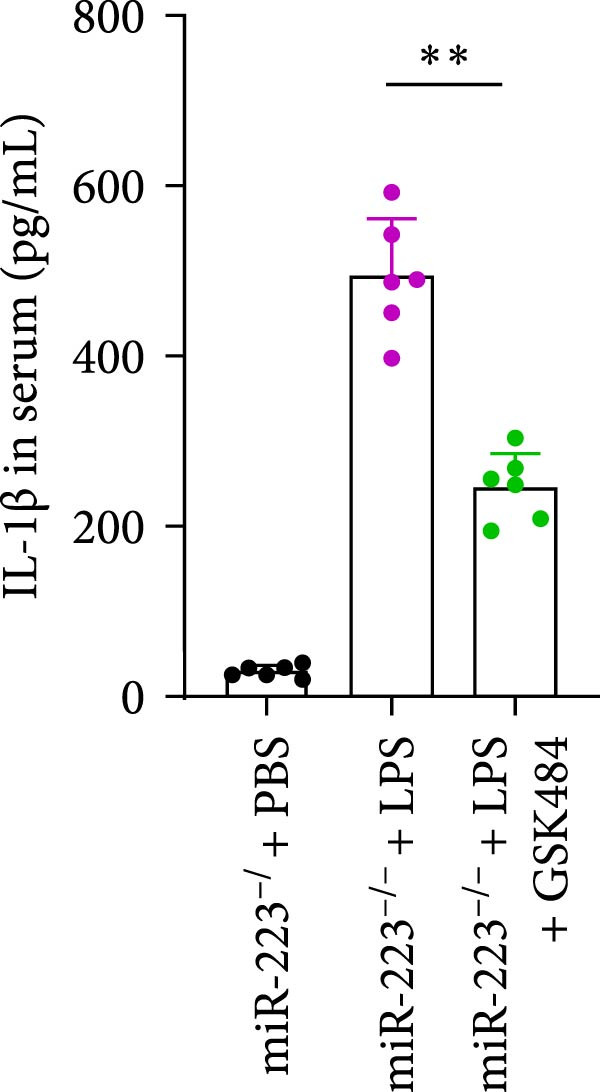
(H)

**Figure:**
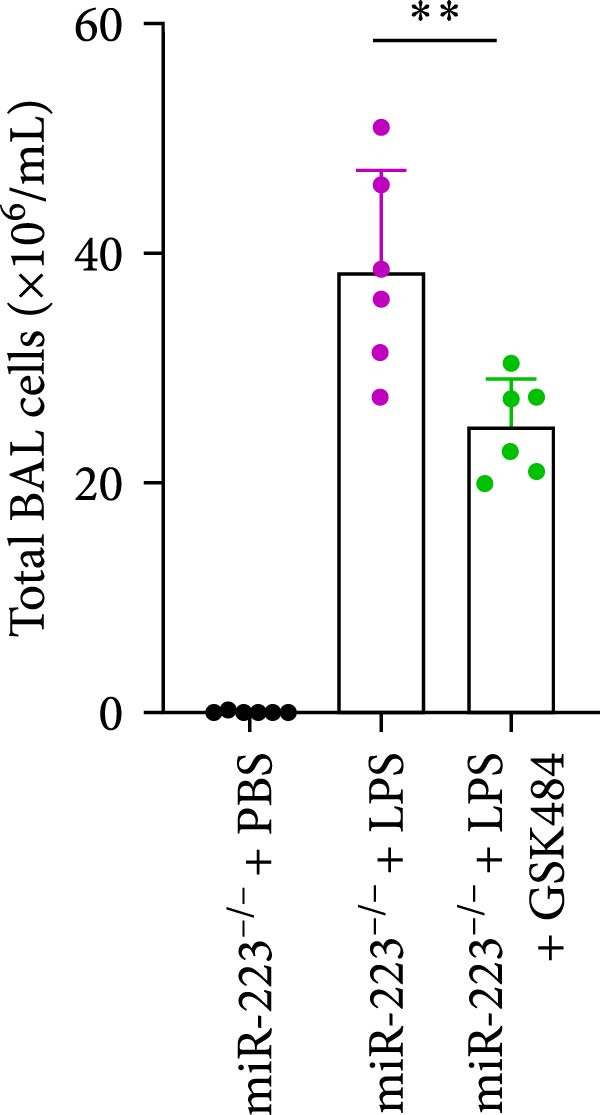
(I)

**Figure:**
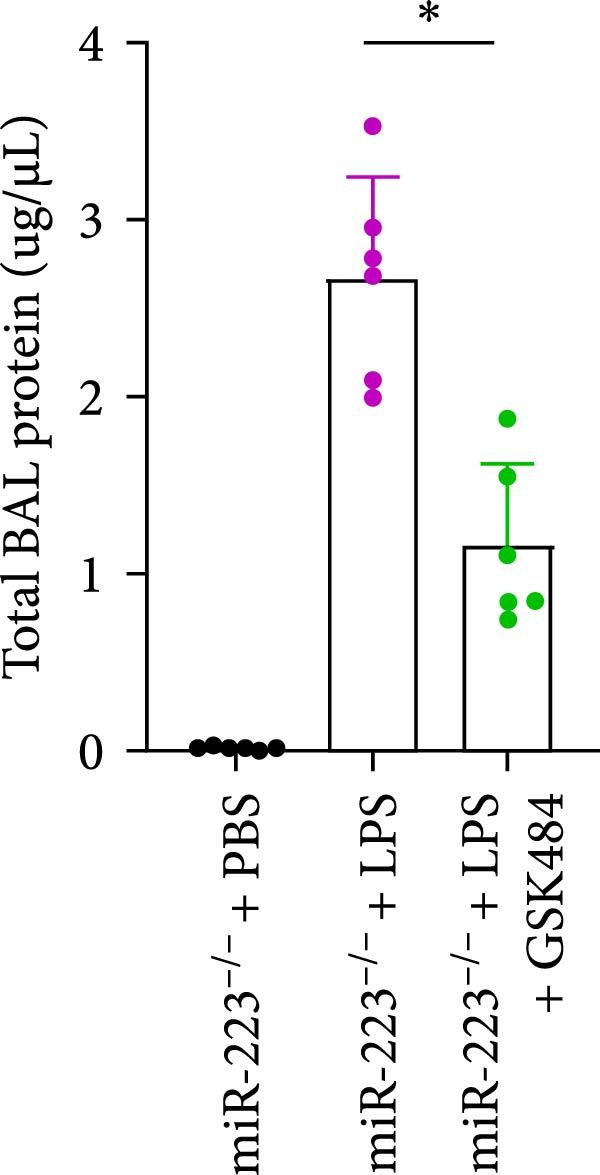
(J)

**Figure:**
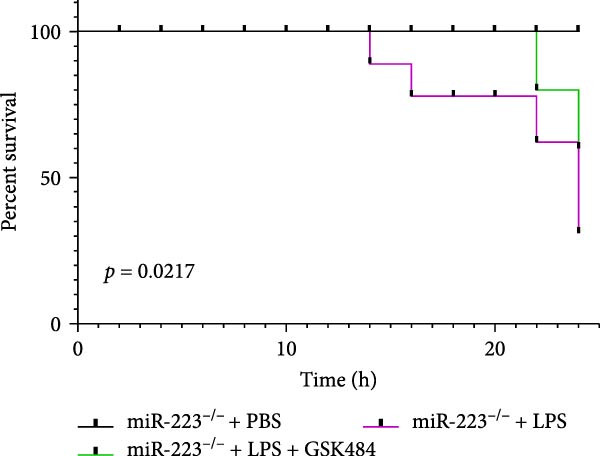
(K)

### 3.6. miR‐223 Regulates NETs Formation and Alveolar Epithelial Cell Injury in a Coculture Model

By establishing a neutrophil–alveolar epithelial cell coculture system, we systematically elucidated the protective mechanism of miR‐223 in ALI. RT‐PCR analysis demonstrated the expression of miR‐223 in HL‐60 cells, compared with LPS treatment, and the LPS + miR‐223 mimic group showed significantly increased miR‐223 expression, while the LPS + miR‐223 inhibitor group exhibited nearly undetectable levels of miR‐223 (Figure 6A). WB analysis demonstrated that transfection with miR‐223 mimics significantly suppressed NE expression (Figure 6B,C). Further mechanistic studies revealed significantly increased expression of H3Cit and MPO in the LPS‐stimulated coculture environment. We observed that miR‐223 deficient neutrophils produced higher levels of H3Cit and MPO (Figure 6D–F), accompanied by markedly increased release of inflammatory factors (TNF‐α, IL‐6, and IL‐1β; Figure 6G–I) in the supernatant and significantly reduced viability of alveolar epithelial cells (Figure 6J). Importantly, this phenomenon could be effectively reversed by the NE inhibitor Sivelestat or the NET formation inhibitor GSK484 (Figure 6J). These findings not only reveal a novel mechanism by which miR‐223 regulates NETosis through targeting NE but also provide potential therapeutic targets for ALI.

Figure 6miR‐223 regulates NETs formation and alveolar epithelial cell injury in a coculture model. (A) RT‐PCR analysis of miR‐223 expression in HL‐60 cells: LPS + miR‐223 mimic group showed significantly increased miR‐223 expression, while LPS + miR‐223 inhibitor group exhibited nearly undetectable levels compared to LPS treatment. (B,C) Western blot analysis demonstrating transfection with miR‐223 mimics significantly suppressed neutrophil elastase expression. (D–F) miR‐223 deficient neutrophils produced higher levels of NET formation markers H3Cit and MPO in LPS‐stimulated coculture environment. (G–I) Supernatant analysis showed markedly increased release of inflammatory factors (TNF‐α, IL‐6, and IL‐1β) in miR‐223 deficient coculture systems. (J) Cell viability assay revealed significantly reduced alveolar epithelial cell viability in miR‐223 deficient coculture, which could be effectively reversed by Sivelestat or GSK484.

**Figure:**
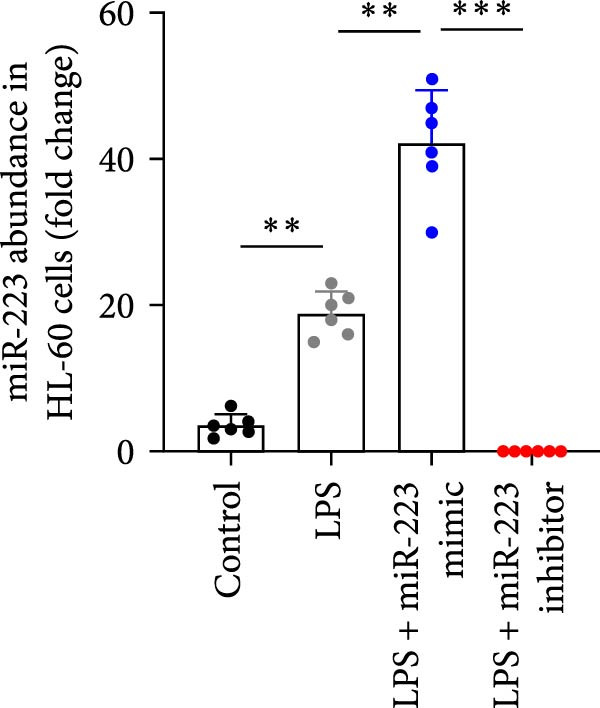
(A)

**Figure:**
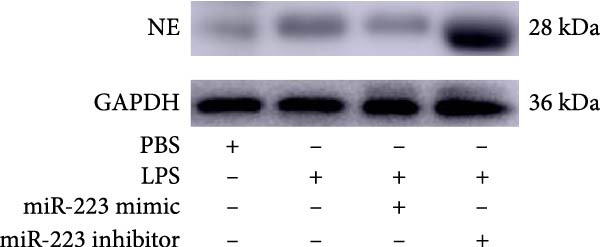
(B)

**Figure:**
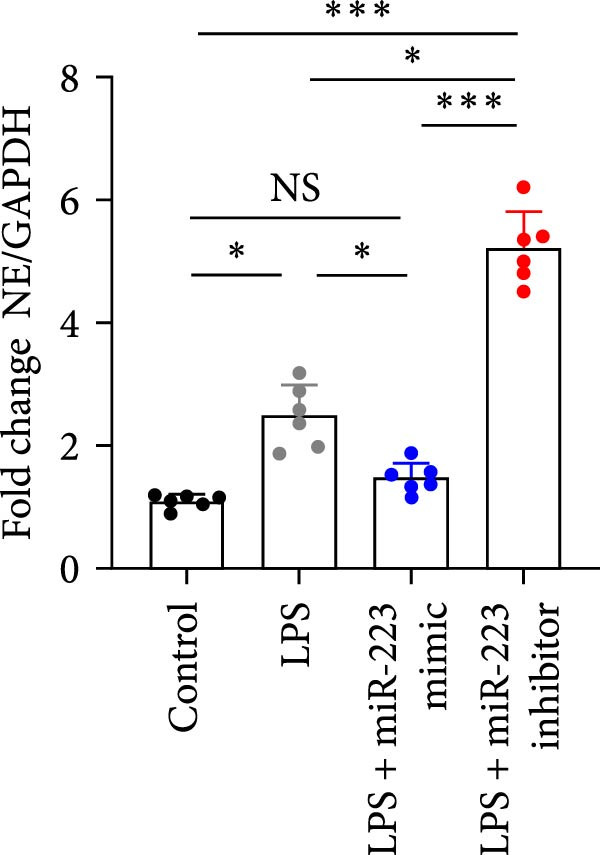
(C)

**Figure:**
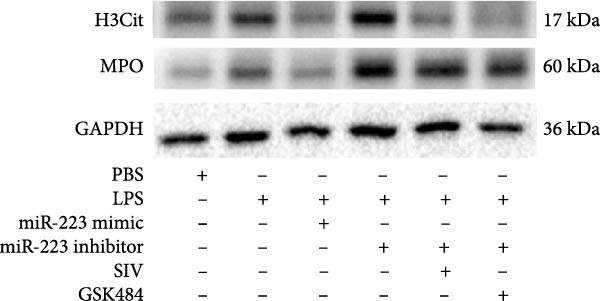
(D)

**Figure:**
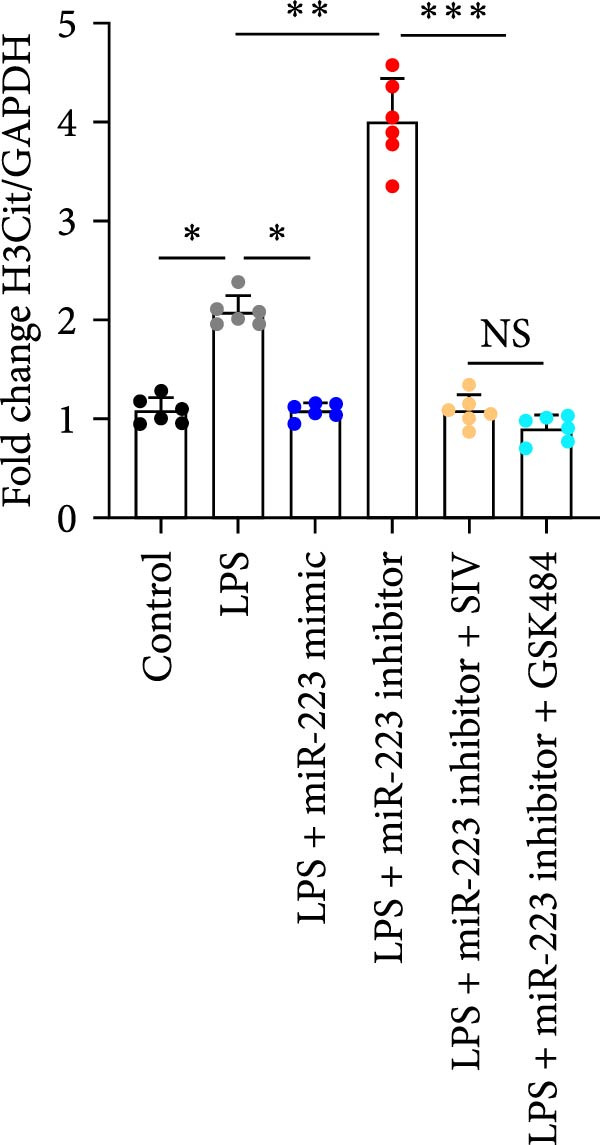
(E)

**Figure:**
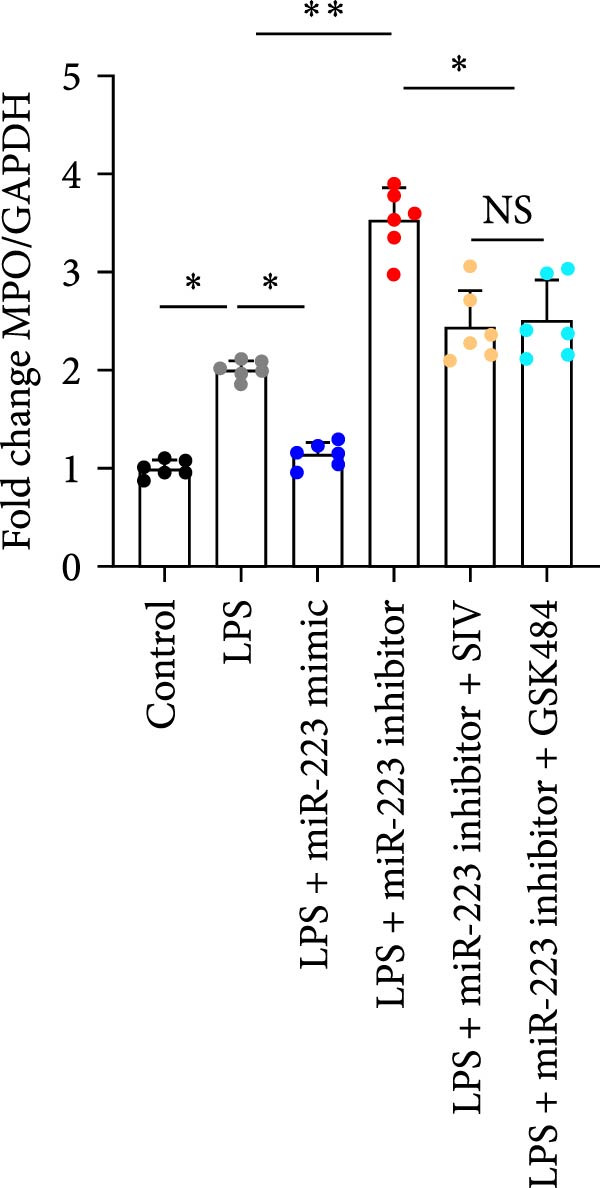
(F)

**Figure:**
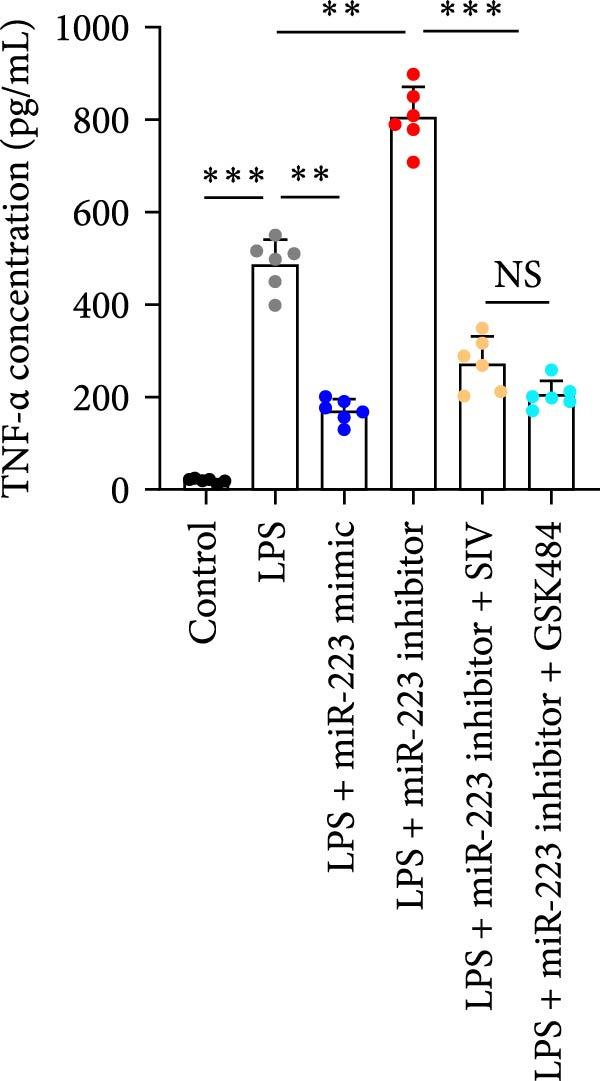
(G)

**Figure:**
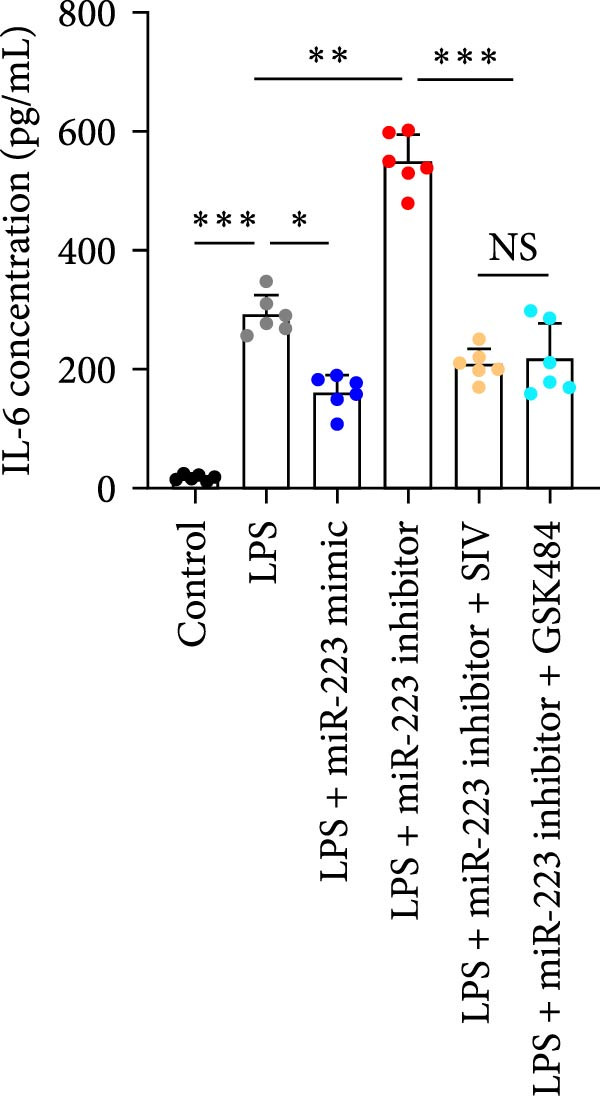
(H)

**Figure:**
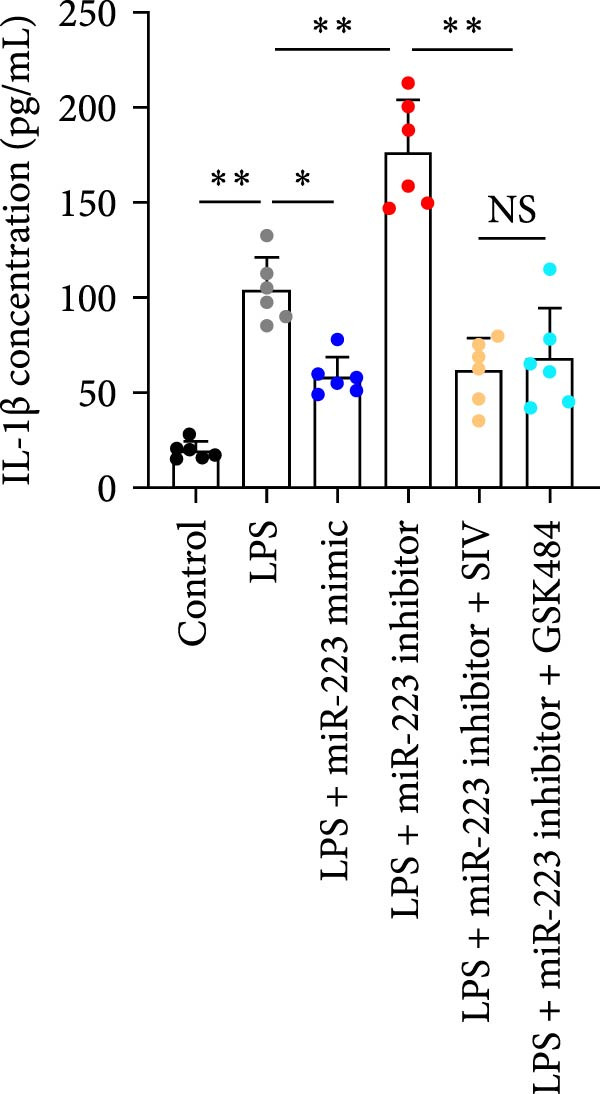
(I)

**Figure:**
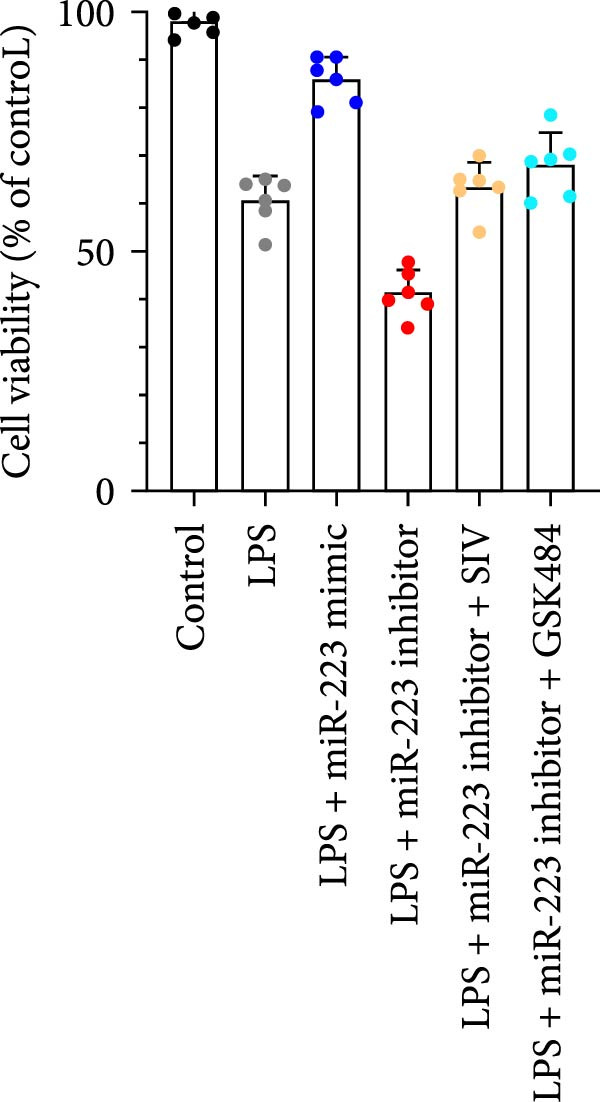
(J)

## 4. Conclusion

**Figure 7:**
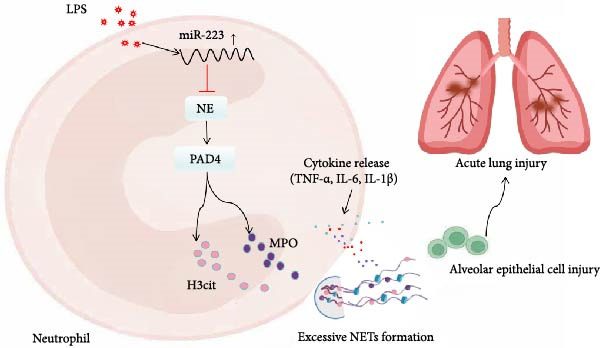
Our proposed model illustrates the central regulatory role of miR‐223 in ALI pathogenesis. The schematic depicts that the deficiency of miR‐223 serves as an upstream event, leading to the overactivation of NE signaling and subsequent excessive formation of NETs. This cascade, in turn, acts as a critical downstream effector, exacerbating pulmonary inflammation and ultimately contributing to the progression of ALI. The diagram integrates these key components to provide a coherent visual summary of the hypothesized pathogenic mechanism.

ALI is characterized by acute diffuse inflammation and dysregulation within the lungs, primarily triggered by various direct and indirect injurious factors, and is closely associated with high morbidity and mortality rates [20]. The pathogenesis of ALI is exceedingly complex, involving multiple biological pathways. Current studies indicate that neutrophils play a critical role in the development of ARDS [21]. NETs, first described as an antibacterial mechanism by Brinkmann et al. [22], are now recognized to contribute to ALI pathogenesis. However, the regulatory mechanisms remain unclear. In this study, we confirmed the involvement of NETs in the pathogenesis of ALI. Specifically, we found that the miR‐223/NE pathway is also involved in the pathological process of ALI, and that the regulation of NETs‐mediated inflammatory lung injury by miR‐223/NE has novelty and clinical application value(Figure 7).

As multifunctional regulatory factors, miR‐223 and NE play distinct roles in modulating the inflammatory response of neutrophils and lung injury. miR‐223, a neutrophil‐enriched miRNA, acts as a fine‐tuner of inflammation by targeting NLRP3, STAT3, and IκB kinase α, thereby suppressing excessive neutrophil activation and NETosis [23–25]. Studies show miR‐223^−/−^ mice exhibit exacerbated ALI due to uncontrolled neutrophilic inflammation, while miR‐223 overexpression attenuates lung damage [26, 27]. Conversely, NE, a serine protease stored in azurophilic granules, promotes IL‐8 secretion and neutrophil recruitment, perpetuating lung injury in ALI/ARDS [28]. Our neutrophil–alveolar epithelial cell coculture experiments confirmed that miR‐223 deficiency significantly enhances NETs formation and inflammatory responses. Conversely, miR‐223 overexpression effectively suppresses these processes. These findings suggest that miR‐223 acts as a critical upstream regulator, likely through a network of target genes. Identifying its direct downstream effectors will be essential to fully elucidate the molecular pathway governing NETosis and inflammation in ALI. Mechanistically, our in vivo and in vitro experiments demonstrate that miR‐223 and NE exert antagonistic effects on NETs formation: NET generation was significantly enhanced both in miR‐223 knockout mice and corresponding cell models, while NE inhibition effectively suppressed this process in both experimental settings. Thus, miR‐223 and NE can be considered key regulatory factors in the formation of NETs in ALI. Our results indicate that the deficiency of miR‐223 promotes the generation of NE in the lungs, thereby enhancing NETs formation and exacerbating lung inflammation and progression of ALI. Notably, miR‐223 indirectly regulates NE activity by modulating neutrophil lifespan and priming. For instance, miR‐223 inhibits NE release by suppressing PKCα‐mediated degranulation, while NE can downregulate miR‐223 in a feedback loop during sustained inflammation [29]. Although NE, a component associated with NETs, is considered an important mediator of ALI, clinical studies validating that Sivelestat can improve the prognosis of ARDS patients are still lacking. However, our experimental results also indicate that the use of the drug inhibitor Sivelestat to block NE significantly alleviated neutrophil infiltration and lung damage in ALI mice.

Moreover, our experiments show that neutrophils are the critical mediating factor for the beneficial effects of miR‐223 in a mouse model of ALI. On one hand, compared to WT mice, neutrophil migration to the lungs is significantly enhanced in miR‐223‐deficient mice under ALI induction; on the other hand, in the absence of miR‐223, the formation of NETs driving lung tissue inflammation and lung injury is significantly increased. Research by Jonathan et al. [13] noted that neutrophils lacking miR‐223 exhibit hyper‐maturation, are highly sensitive to activation stimuli, and demonstrate enhanced bactericidal activity, leading to spontaneous inflammatory lung pathology in miR‐223‐deficient mice and significant lung tissue damage after LPS stimulation [30]. These data further support miR‐223 as a fine‐tuning factor in neutrophil generation and inflammatory response.

NETs are complex structures composed of DNA, histones, elastase, and other components, which play a role in clearing microorganisms. However, abnormal activation of NETs is involved in various pulmonary immune inflammatory diseases [31]. Although the formation of NETs has been observed in ARDS, the regulatory mechanisms remain unclear. To explore their function, this study employed an LPS‐induced ALI mouse model, and the results showed that NETs blockade mediated by GSK484 significantly reduced neutrophil infiltration in the lungs, suggesting that LPS‐induced NETs may exacerbate neutrophil infiltration in the lungs through a positive feedback mechanism. Nevertheless, compared to the WT controls, the beneficial effects of GSK484 pretreatment in miR‐223^−/−^ mice were more pronounced, primarily due to the increased severity of lung injury in miR‐223^−/−^ mice under the baseline conditions of vehicle treatment. This mechanism was further corroborated by our in vitro coculture experiments. Recent studies have found that NETs have broad potential clinical applications in the diagnosis and treatment of ARDS, and blocking the expression of NETs may be a new approach to ARDS treatment [32, 33]. Existing evidence suggests that in various inflammation‐related pathological states, pharmacological inhibition of NETs formation can significantly mitigate tissue damage, including sepsis, ischemia‐reperfusion injury, and acute kidney injury [34–36]. Clearly, numerous factors that play important regulatory roles in NETs formation and NETs‐induced inflammation are potential therapeutic targets for ALI. Notably, although our research results did not fully reverse ALI and its associated mortality, interventions through miR‐223 gene knockout or the use of drugs (such as GSK484 or Sivelestat) effectively or partially inhibited inflammation and lung injury. This finding emphasizes the necessity for more potent and rapidly acting pharmacological therapies targeting NETs for the treatment of ALI.

However, this research has certain limitations: the specific molecular mechanisms by which miR‐223 regulates neutrophils remain unclear and require further investigation of its downstream targets; while NETs inhibition shows promising results in preclinical models, clinical validation in ARDS patients is still needed.

The results of this study conclusively demonstrate the pivotal role of the miR‐223/NE/NETs regulatory axis as a key factor regulating neutrophil infiltration, playing an important role in inflammation mediated by NETs, thereby exacerbating neutrophil infiltration and promoting the progression of ALI. These findings indicate that therapeutic interventions targeting the regulation of miR‐223/NE and NETs may provide a new theoretical basis for the further optimization of ALI treatment.

## Conflicts of Interest

The authors declare no conflicts of interest.

## Funding

The study was supported by the Hunan Provincial Natural Science Foundation of China (Grant 2025JJ50739).

## Data Availability

The data that support the findings of this study are available from the corresponding author upon reasonable request.
